# Design of a parallel cluster-randomized trial assessing the impact of a demand-side sanitation and hygiene intervention on sustained behavior change and mental well-being in rural and peri-urban Amhara, Ethiopia: *Andilaye* study protocol

**DOI:** 10.1186/s12889-019-7040-6

**Published:** 2019-06-21

**Authors:** Maryann G. Delea, Jedidiah S. Snyder, Mulusew Belew, Bethany A. Caruso, Joshua V. Garn, Gloria D. Sclar, Mulat Woreta, Kassahun Zewudie, Abebe Gebremariam, Matthew C. Freeman

**Affiliations:** 1 Department of Environmental Health, Rollins School of Public Health, Emory University, Atlanta, GA USA; 2 Emory Ethiopia, Bahir Dar and Addis Ababa, Ethiopia; 3 School of Community Health Sciences, University of Nevada, Reno, NV USA

**Keywords:** Water, sanitation, and hygiene, WASH, Impact evaluation, CLTSH, Behavior change, Behavioral maintenance, Evidence-based intervention, NTDs, Demand-side sanitation and hygiene

## Abstract

**Background:**

Unimproved water, sanitation, and hygiene (WASH) behaviors are key drivers of infectious disease transmission and influencers of mental well-being. While WASH is seen as a critical enabler of health, important knowledge gaps related to the content and delivery of effective, holistic WASH programming exist. Corresponding impacts of WASH on mental well-being are also underexplored. There is a need for more robust implementation research that yields information regarding whether and how community-based, demand-side interventions facilitate progressive and sustained adoption of improved sanitation and hygiene behaviors and downstream health impacts. The purpose of this protocol is to detail the rationale and design of a cluster-randomized trial evaluating the impact of a demand-side sanitation and hygiene intervention on sustained behavior change and mental well-being in rural and peri-urban Amhara, Ethiopia.

**Methods:**

Together with partners, we developed a theoretically-informed, evidence-based behavioral intervention called *Andilaye*. We randomly selected and assigned 50 sub-districts (*kebeles*) from three purposively selected districts (*woredas*); half to receive the *Andilaye* intervention, and half the standard of care sanitation and hygiene programming (i.e., community-led total sanitation and hygiene [CLTSH]). During baseline, midline, and endline, we will collect data on an array of behavioral factors, potential moderators (e.g., water and sanitation insecurity, collective efficacy), and our primary study outcomes: sanitation and hygiene behaviors and mental well-being. We will perform a process evaluation to assess intervention fidelity and related attributes.

**Discussion:**

While CLTSH has fostered sanitation and hygiene improvements in Ethiopia, evidence of behavioral slippage, or regression to unimproved practices in communities previously declared open defecation free exists. Other limitations of CLTSH, such as its focus on disgust, poor triggering, and over-saturation of Health Extension Workers have been documented. We employed rigorous formative research and practically applied social and behavioral theory to develop *Andilaye*, a scalable intervention designed to address these issues and complement existing service delivery within Ethiopia’s Health Extension Program. Evidence from this trial may help address knowledge gaps related to scalable alternatives to CLTSH and inform sanitation and hygiene programming and policy in Ethiopia and beyond.

**Trial registration:**

This trial was registered with clinicaltrials.gov (NCT03075436) on March 9, 2017.

**Electronic supplementary material:**

The online version of this article (10.1186/s12889-019-7040-6) contains supplementary material, which is available to authorized users.

## Background

### Study rationale

Inadequate water, sanitation, and hygiene (WASH) are key drivers of infectious disease transmission and result in adverse mental and social well-being [1–4]. Diarrhea accounts for an estimated 1.4 million deaths annually [5, 6] and nearly 20% of all under-5 deaths in low-income settings [7]. Deficiencies in WASH are also a major contributor of neglected tropical diseases (NTDs) [8, 9]. Over one billion people are at risk of soil-transmitted helminthiasis, which leads to nearly five million disability adjusted life years (DALYs), and schistosomiasis leads to a further two million DALYs [10, 11]. Trachoma, the leading infectious cause of blindness [12], is precipitated by repeated infection with bacteria, which is often perpetuated by poor hygiene [13]. These infections are environmentally mediated [14], and are largely attributed to inadequate WASH [15, 16].

Many WASH programs focus only on measures of infectious diseases or growth of young children to assess programmatic impact and public health relevance. This narrow focus is at odds with the World Health Organization (WHO)‘s definition of health as “a state of complete physical, mental, and social well-being and not merely the absence of disease or infirmity” [17]. Poor WASH conditions are associated with adverse mental health outcomes [3]. Stress and depression, particularly amongst women, are linked with poor sanitation access [18]. Improvements in women’s mental health, however, would likely require more than physical access to sanitation facilities, and may also entail changes in gendered norms that contribute to women’s sanitation insecurity [19]. Evidence also suggests associations between water and sanitation insecurity and mental well-being [20, 21], though most data focusing on the role of WASH in improving social and mental well-being is observational [3]. Quantifying the relationships between WASH improvements and these non-traditional outcomes and impacts can inform programs and policies that facilitate health equity.

While WASH-related research generally focuses on the individual, communities are often a more critical point of investigation. Due to the dynamics of infectious disease transmission, the effects of WASH interventions often depend on community level coverage and uptake [22]. Financing and maintenance of certain water and sanitation interventions may also depend on minimum levels of social capital and social cohesion [23–26]. Evidence suggests that correct, consistent, and sustained uptake of WASH interventions—other critical drivers of protective effects—depend in part on a variety of social constructs, such as collective efficacy, descriptive and social norms, and social identity [27, 28]. Nevertheless, research on these inter-personal behavioral factors, their influence on collective WASH behaviors, and health and development effects is comparatively limited. Consequently, there is inadequate evidence about how these behavioral factors should be considered in developing, implementing, and evaluating WASH programming and policy.

### Ethiopia context

Despite steady reductions in open defecation since 2000, Ethiopians have some of the poorest access to basic water and sanitation, and one of the highest levels of access inequity [29]. The Ethiopia Federal Ministry of Health’s (FMoH) Health Extension Program (HEP), and its accompanying community-led total sanitation and hygiene (CLTSH) module, represent government-backed, low-cost, and locally acceptable approaches for improving sanitation and hygiene. CLTSH was originally implemented in Ethiopia through a partnership between the Amhara Regional Health Bureau (ARHB), the USAID-funded Hygiene Improvement Project (HIP), and the Water and Sanitation Program (WSP) in 2006. While an evaluation of CLTSH demonstrated a decrease in open defecation during 2008 to 2010, and an increase in unimproved latrine utilization from 19 to 46%, there was no evidence of change in coverage of improved sanitation facilities [30]. The Health Extension Services Package being delivered via the HEP, and its accompanying CLTSH module, are currently being scaled throughout Ethiopia [31].

### Objective, study aim, research question and hypothesis

The objective of this study protocol is to provide a rationale for and details related to the design of our two-arm, parallel group cluster-randomized trail (CRT) evaluating the impact of an intervention designed to specifically address documented limitations of the CLTSH approach. The aim of the *Andilaye* Trial - Amharic for “togetherness/integration” - is to use behavioral theory and evidence from formative research to inform the design of a novel holistic community-based WASH intervention and evaluate its impact on sustained behavior change and mental well-being. The study’s primary research question is: To what extent can *Andilaye’s* enhanced, demand-side sanitation and hygiene intervention impact sustained behavior change and mental well-being, above and beyond CLTSH? We hypothesize that individuals in communities randomized to receive the *Andilaye* intervention are more likely to sustainably adopt improved sanitation and hygiene behaviors and demonstrate gains in mental well-being compared to individuals in communities randomized to the standard of care CLTSH programming.

This study is designed to generate evidence to address knowledge gaps related to demand-side sanitation and hygiene programming and examine less studied, yet critical, inter-personal factors related to sustained behavioral adoption and downstream health impacts. Data from the *Andilaye* Trial will be used to: (1) identify ways in which WASH-related behavior change components can be mainstreamed into the FMoH’s HEP; (2) determine the effectiveness of an enhanced holistic demand-side sanitation and hygiene intervention that promotes NTD-preventive improved WASH behaviors; (3) investigate whether changes in personal hygiene and sanitation behaviors are sustained, and which factors contribute to this; (4) document the cost-effectiveness of integrated, holistic WASH behavior change promotion; and (5) assess whether collective efficacy and water and sanitation insecurity modify intervention effectiveness.

## Methods

### Trial design

Emory University and its consortium partners are conducting a two-year impact evaluation, designed as an ex-ante two-arm, parallel CRT. Clusters are defined as rural or peri-urban *kebeles* from three *woredas* in West Gojjam and South Gondar Zones, Amhara National Regional State, Ethiopia. As indicated in further detail below, we randomly selected and assigned clusters; half to receive the *Andilaye* intervention, half to receive the current standard of care sanitation and hygiene programming (i.e., interventions related to FMoH’s existing CLTSH programming). The intervention arm will receive *Andilaye*, a demand-side sanitation and hygiene intervention informed by social and behavioral theory and empirical evidence, particularly evidence generated during *Andilaye’s* formative research phase. The *Andilaye* intervention promotes selected, improved WASH behaviors and constituent practices deemed locally appropriate for inclusion in a demand-side intervention. The intervention focuses on positive, community-oriented motivators of behavioral change, promotes achievable incremental improvements, and incorporates strategies that facilitate behavioral maintenance (i.e., prevention of behavioral slippage or relapse back to unimproved behaviors). We designed the *Andilaye* intervention to integrate behaviors that could contribute to improvements in mental and social well-being and the control of infectious diseases, including trachoma and soil-transmitted helminthiasis. The control arm (counterfactual comparator) will receive FMoH’s existing CLTSH programming, and no attempt will be made to modify the government’s roll-out of these interventions or the Health Extension Services Package. The study team will work with government partners to minimize contamination from other WASH interventions in our study sites, to the greatest extent possible.

We will conduct a process evaluation alongside our impact evaluation to assess intervention implementation fidelity, participation and dose response, and contextual factors. The purpose of the process evaluation is to determine the quality and integrity of the *Andilaye* intervention, as implemented; the extent to which participant engagement and dose response are associated with the dose delivered; and the social, political, and economic factors that may influence intervention implementation. We will take a mixed methodological approach to collect process data over the *Andilaye* study period. We will collect quantitative data via structured household-level surveys, activities observations, post-training assessments, and systematic audits of records. We will collect qualitative data via semi-structured interviews and informal discussions with key informants (e.g., government stakeholders, Health Extension Workers [HEWs], Women’s Development Army Leaders [WDALs], and community members at large) and participant observations during relevant trainings and intervention activities. These data will allow us to better interpret our impact evaluation data and determine how and why change did or did not occur (e.g., via the identification of potential barriers to and facilitators of intervention uptake).

### Timeline

**Fig. 1:**
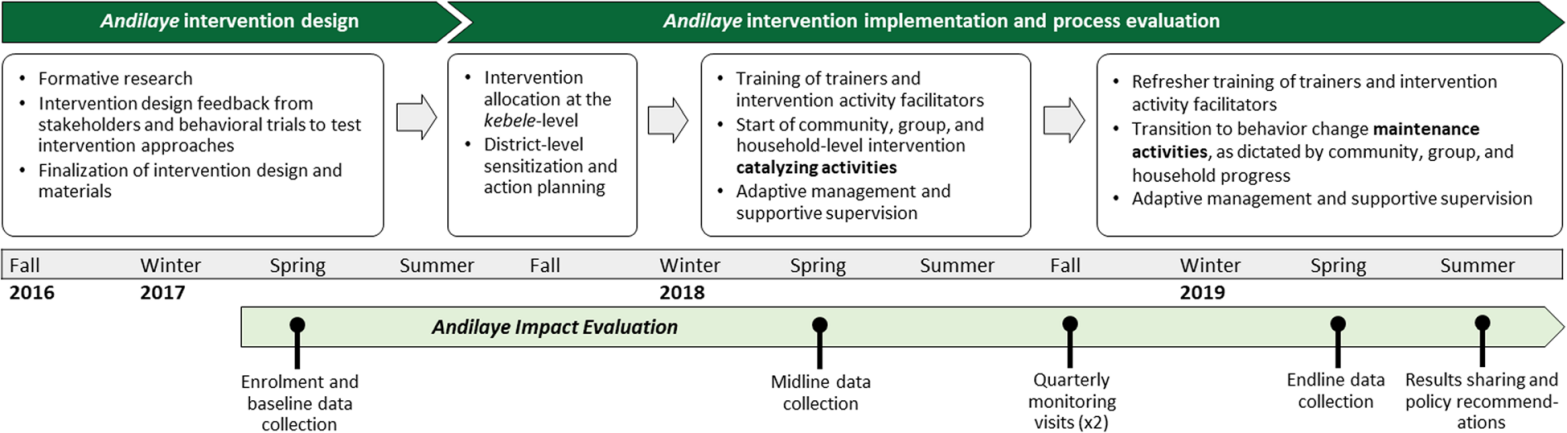
Timeline of *Andilaye* study

The *Andilaye* Trial consists of three major phases: (1) formative research and intervention design, (2) intervention implementation and process evaluation, and (3) impact evaluation (Fig. 1). Formative research and intervention design were conducted during September 2016 to February 2017. *Kebele* and household enrollment took place during baseline data collection – March to April 2017. Implementation of *Andilaye* intervention activities began in September 2017 and will continue, with a focus on behavior change catalyzing activities, through midline data collection – March to April 2018. Intervention activities will transition to activities focused on behavioral maintenance, as dictated by community, group, and household-level progress, through to endline evaluation – March to May 2019.

### Study setting

**Fig. 2:**
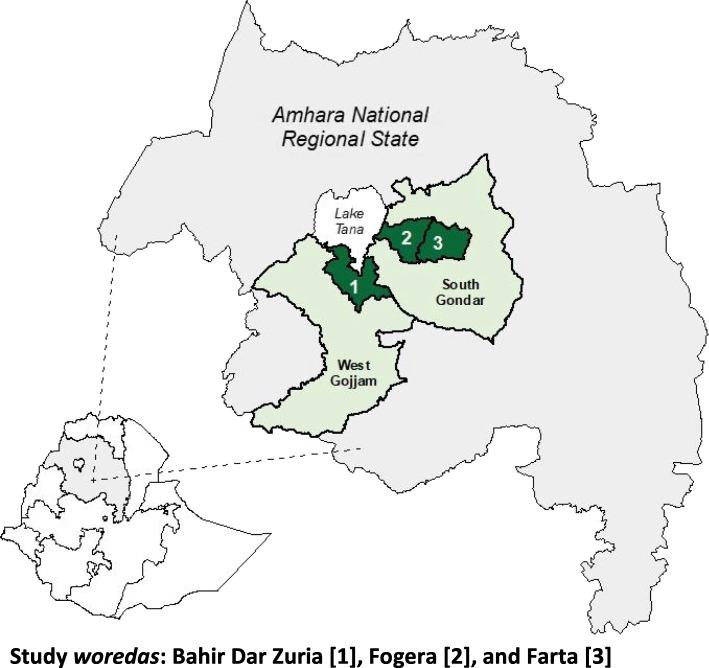
Map of three *woredas* in Ethiopia targeted for the *Andilaye* Trial. *Map generated using publicly available shape files*

The *Andilaye* Trial is being carried out in Amhara National Regional State, a region of Ethiopia in which WASH conditions are poor [32], behavioral slippage has been documented, and several NTDs are hyperendemic [33]. As with the rest of Ethiopia, where CLTSH is being scaled nationally, study communities have either been triggered with CLTSH or are scheduled for triggering in the near future. Three districts - Bahir Dar Zuria *Woreda* in West Gojjam Zone and Fogera and Farta *Woredas* in South Gondar Zone - were targeted for this study given they represent a range of the topographical conditions present in Amhara, and Ethiopia in general (Fig. 2).

### Andilaye intervention

#### Design process

We employed a rigorous intervention design approach that leveraged social and behavioral theory, formative research results, intervention mapping [34], and inputs from Ethiopian stakeholders in the health and development sectors to ensure the *Andilaye* intervention was contextually appropriate and technically sound. Formative research was grounded in several behavioral theories and frameworks, including the *Capability, Opportunity, Motivation, and Behavior* (COM-B) model [35], the *Theory of Triadic Influence* [36], and the *Risks, Attitudes, Norms, Abilities, and Self-Regulation (RANAS)* approach [37]. *Kebeles* in which formative research activities were conducted were similar to *kebeles* eligible for the *Andilaye* Trial; however, in most circumstances, the *kebeles* were not deemed eligible for inclusion in the study given their involvement in the formative research. Prior to finalizing the design of the content and implementation strategies employed by the *Andilaye* intervention, we conducted a month-long behavioral trial (i.e., trial of improved practices) in formative research communities to provide an opportunity for targeted change agents and intervention recipients to provide their feedback regarding intervention functions (e.g., delivery modalities and intervention materials). The finalized *Andilaye* intervention addresses issues related to the over-extension of HEWs and over-saturation of messaging via the HEP’s Health Extension Services Package through the engagement of WDALs and additional community change agents as mechanisms for intervention delivery. The intervention leverages recent work conducted by the World Bank, and incorporates feedback from relevant stakeholders, including FMoH, ARHB, Zonal Health Departments, *Woreda* Health Offices, other non-governmental organizations, and individuals such as formative research community members.

#### Intervention details

*Andilaye’s* intervention motto, “*Together we can be a strong, caring, healthy community*”, and related intervention components offer aspirational messages that emphasize the need for collective action to make positive change in the community and use verbal persuasion to enhance collective efficacy perceptions. The intervention focuses on three behavioral themes, informed by formative research: (1) sanitation, (2) personal hygiene, and (3) household environmental sanitation. Within these themes are 11 constituent practices targeted by the intervention; these practices were identified through formative research and the intervention design process as ones that could be targeted using demand-side approaches, and were seen as achievable, per stakeholder feedback (Additional file 1: Figure S1). We emphasize that behaviors usually represent a constellation of practices [38], and while the *Andilaye* intervention promotes 11 constituent practices of interest, these practices represent only three improved WASH behaviors. As such, our intervention actually focuses on fewer practices than many WASH interventions while also clearly specifying all necessary practices required to adopt the related improved behaviors.

#### Target audiences

While primary caregivers of the study’s index children (i.e., youngest child in the study household aged 1–9 years at baseline) comprise the intervention’s primary target audience, we designed the intervention to address inter-personal behavioral factors and promote behavior change among all household members and the community at large. The logic model presented in Additional file 1: Figure S1 reflects the summarized theory of change in which the *Andilaye* intervention is grounded. *Andilaye* intervention activities operate at four levels – district, community, group, and household (Additional file 2: Table S1) – and employ a variety of behavior change and maintenance techniques. Related intervention components leverage several motives to address behavioral antecedents and determinants at various levels of influence. Behavioral antecedents are precursors that need to be addressed before behavioral change and maintenance can occur. These include psychosocial factors such as attitudes and normative beliefs regarding improved practices, perceived and actual abilities to perform improved practices, self-regulation, and intentions to initiate and maintain the adoption of improved practices. Behavioral determinants reflect physical and contextual conditions, such as water availability and facilities access, that mediate the adoption and translation of behaviors into the execution of improved practices.

#### Intervention activities

Prior to activity roll out in the community, the *Andilaye* intervention commences with district-level capacity building activities, such as action planning and training of trainers and intervention activity facilitators. Further, district-level refresher trainings and adaptive management activities are conducted to reinforce previously acquired knowledge and skills, address trainer/facilitator turnover, and review successes and address challenges faced in implementing group and household level activities. Community-level activities include the ‘Whole System in the Room’ [39] (Additional file 2: Table S1), community mobilization and commitment events, and cross-fertilization visits. These activities are intended to engage community stakeholders in action planning, create an enabling environment in which change may occur, and address inter-personal factors related to public commitment, social norms, and social support related to improved practices, among others. Group-level activities such as structured community conversations, provide further opportunity for peer-to-peer counselling and support. These activities serve to address action knowledge and capacity, enhance barrier identification and planning, shift perceptions regarding empirical expectations, and improve perceptions regarding individual and community capabilities (e.g., self-and collective efficacy appraisals). Household-level counselling visits by WDALs provide personalized counselling to caregivers to equip them with the knowledge, skills, and motivation necessary (e.g., individual and household goal setting and monitoring [self-regulation], self-efficacy, tailored barrier identification and planning) to adopt and maintain improved WASH practices.

### Study outcomes and measures

Primary outcomes of interest include:

1. Sustainability of WASH-related behaviors, as measured through the proportion of individuals and households consistently practicing improved targeted WASH behaviors, including: (1) sanitation, (2) personal hygiene, and (3) household environmental sanitation, more specifically, the 11 constituent practices identified above; and
2. Mental well-being, as measured through standardized mental well-being scale scores generated via the administration of the WHO-5 [40] and Hopkins Symptom Checklist (HSCL) [41].

Secondary outcomes of interest include:

- Short-term behavioral outcomes, measured as the proportion of households with improved (private or shared) latrines and washing facilities that are functional and available for use; proportion of households using functional latrines and washing facilities; proportion of households with all members exclusively using a latrine for defecation; proportion of households disposing of child feces in an improved latrine; proportion of households with all children in the household with a clean face and hands;
- Intermediate behavioral antecedents, measured as the proportion of households with improved knowledge regarding the implications of improved WASH practices (i.e., perceptions regarding negative externalities); proportion of households that indicate positive attitudes, perceptions toward improved sanitation and good hygiene practices; change in normative expectations related to open defecation, exclusive latrine use for defecation, and personal hygiene practices;
- Diarrhea period prevalence, as measured through caregiver self-report;
- Sanitation insecurity, as measured through changes in sanitation insecurity scale scores [19];
- Collective efficacy measures, as measured through changes in collective efficacy scale scores [42]; and
- Water insecurity, as measured through changes in water insecurity scale scores [43].

### Sample size

We powered this study on mental well-being outcomes, as measure by the HSCL [41], which reflected the most restrictive primary outcomes in terms of required sample size. Various studies from Ethiopia and East Africa suggest that approximately 20–35% of rural women experience elevated symptoms of common mental disorders such as anxiety and depression [44, 45]. Drawing on two studies that have used the HSCL in East Africa [41], we estimated that average scores generated by this tool would be around 1.5 (SD 0.5). Using unpublished data from a large on-going study of young people in Ethiopia [46], we estimated the intra-cluster correlation (ICC) for a measure of mental health was approximately 0.05, although we suspected this may be low given the sample consists of young people, and the measure captures more severe mental health symptoms. Unpublished data from rural households in South Omo, Ethiopia are approximately 33% higher on a common mental health scale; suggesting that water insecurity is linked to mental health, and the effect is appreciable [47]. There are limited data on the impact of changes in WASH on mental well-being, so we used a similar difference to estimate our impact. Our sample size determination (Additional file 3: Table S2) indicated that we should recruit and enroll a total of 25 households from each of 50 study clusters (25 clusters per study arm). We increased our final sample size to accommodate for 20% of households being lost to follow-up. Our target sample, therefore, included 30 households in each *kebele* study cluster, or 1500 households in total (i.e., 750 per study arm).

Following baseline, we conducted an ex-post power calculation on our main outcome. Given the prevalence of poor well-being (33.2% - Table 1), and a calculated ICC of 0.026, lower than expected, we determined that we were well powered (> 99%) to detect our original expected 37% relative reduction (an absolute difference of 12% points). Assuming 80% power, the absolute detectible difference is 8% points (a 24% relative reduction). For our other health outcomes, we conducted ex-post power calculations, and determined that we are able to detect a: 7%-point reduction in anxiety, with baseline of 29.7% (ICC: 0.007); 6%-point reduction in depression, with baseline of 20.8% (ICC: 0.01); and 5%-point reduction in emotional distress, with baseline of 17.3% (ICC: 0.005). Given the low prevalence of diarrhea (Table 1) and recent large-scale studies that showed mixed effects of the impact of WASH on diarrhea [48, 49], we considered this a secondary outcome. Given a baseline reported diarrheal prevalence of 9.1% for the index child during the past 7 days (ICC: 0.077), we are powered to see a 6%-point reduction in diarrhea outcomes.

**Table 1:** Baseline data

Indicator	Overall		Intervention		Control	
Demographic information	N	%	N	%	N	%
Respondent was female	1589	90.7	793	91.3	796	90.1
Respondent was mother of index child	1589	84.6	793	85.4	796	83.9
Primary caregiver/mother has at least secondary education	1589	12.8	793	11.9	796	13.8
Primary caregiver/mother is married	1587	89.3	792	91.2	795	87.4
Head of household has at least some secondary education	1579	16.7	791	15.2	788	18.2
Demographic information	N	mean (SE)	N	mean (SE)	N	mean (SE)
Respondent’s age	1589	33.5 (0.38)	793	33.7 (0.52)	796	33.3 (0.55)
Head of household age	1589	41.3 (0.46)	793	41.6 (0.54)	796	41.1 (0.73)
Number of household members	1589	5.3 (0.08)	793	5.3 (0.10)	796	5.3 (0.12)
Household latrine coverage	N	%	N	%	N	%
Households with access to at least one household latrine	1589	65.5	793	64.1	796	66.8
Households with access to an improved household latrine ^a^	1553	39.8	775	39.9	778	39.7
Households with access to a fully constructed household latrine	1583	30.7	792	29.6	791	31.9
Household latrine operation and maintenance	N	%	N	%	N	%
Household has added or improved anything on the latrine since its original construction	1028	12.9	504	11.9	524	13.9
Household latrine characteristics	N	%	N	%	N	%
Presence of drop hole cover in the latrine	1033	13.4	505	12.5	528	14.2
Among those with a drop hole, a cover was situated over drop hole	138	66.7	63	65.1	75	68.0
Defecation practices	N	%	N	%	N	%
Respondent’s primary place of defecation was in the open during last 2 days	1589	37.5	793	39.5	796	35.6
Respondent defecated in any latrine during last 2 days	1589	45.6	793	46.0	796	45.1
Child feces were safely disposed of during the last 2 days	961	40.2	463	38.9	498	41.4
Sharing of household latrine facilities	N	mean (SE)	N	mean (SE)	N	mean (SE)
Given household has a household latrine, number of people from another household who used this latrine during last 7 days, exclusive of household members	1037	0.94 (0.12)	506	1.08 (0.18)	530	0.79 (0.14)
Animal husbandry / other household sanitation practices	N	%	N	%	N	%
Animal feces/waste not left out in open in compound	1589	44.1	793	42.0	796	46.2
Facial cleanliness among children aged 1–9 years	N	%	N	%	N	%
Ocular discharge present	1944	40.3	932	42.2	1012	38.6
Wet nasal discharge present	1944	47.3	932	47.6	1012	46.9
Dry nasal discharge present	1944	65.4	932	64.7	1012	66.1
Dirt/dust/other debris present	1944	69.9	932	68.7	1012	71.0
	N	mean (SE)	N	mean (SE)	N	mean (SE)
Number of times a fly land on the index child’s face during a 1 min observation	1382	4.2 (0.23)	669	4.1 (0.34)	713	4.3 (0.32)
Household washing station coverage	N	%	N	%	N	%
Household hand or facewashing station(s)	1589	78.9	793	77.1	796	78.8
Handwashing practices	N	%	N	%	N	%
The last time the respondent washed, s/he used soap/ash/soapy water	1588	36.4	793	35.1	795	37.7
The last time the respondent defecated, s/he cleaned hands with water and soap, substitute	1585	37.2	791	36.3	794	38.0
The last time the respondent prepared food, s/he cleaned hands with water and soap, substitute before beginning food preparations	1586	39.7	791	41.0	795	38.5
Diarrhea among index children	N	%	N	%	N	%
During the last 2 days, including today, index child had three or more loose stools per day	1577	6.3	782	5.6	795	6.9
During the last 7 days, including today, index child had three or more loose stools per day	1575	9.1	778	8.1	797	10.0
Anxiety and depression	N	% Mean (SD)	N	% Mean (SD)	N	% Mean (SD)
Anxiety ^b^	1584	29.7 1.56 (0.62)	790	29.6 1.56 (0.60)	794	29.7 1.56 (0.63)
Depression ^b^	1588	20.8 1.46 (0.52)	793	21.3 1.45 (0.51)	795	20.4 1.46 (0.52)
Emotional distress ^b^	1583	17.3 1.38 (0.48)	790	17.2 1.38 (0.47)	793	17.4 1.38 (0.48)
*WHO-5 well-being*	N	% Mean (SD)	N	% Mean (SD)	N	% Mean (SD)
Poor well-being ^c^	1586	33.2 16.0 (7.0)	792	31.1 15.6 (7.1)	794	35.3 16.3 (6.8)

^a^ "Improved" based on the WHO/UNICEF Joint Monitoring Programme (JMP) for Water Supply and Sanitation definition. ^b^ We asked respondents to indicate how much the symptoms bothered them in the previous week with four potential response options (not at all [1] to extremely [4]). The first ten symptoms assess anxiety (i.e., ‘suddenly scared for no reason’, ‘nervousness or shakiness inside’), the next 13 assess depression (i.e., ‘feeling low in energy’, ‘feeling hopeless about the future’), and the 23 collectively assess non-specific emotional distress. For each outcome, the score is the sum of the responses divided by the number of items. Each of these scores was dichotomized, with scores greater than 1.75 indicating a positive status for any of the three outcomes. ^c^ We asked respondents about well-being, and responses ranged from ‘(0) At no time’ to (5) All of the time’. Scores were summed, and range from 0 to 25; the higher the score, the better the well-being. Each of these scores was dichotomized with scores below 13 indicating poor well-being

### Eligibility criteria

Rural and peri-urban *kebeles* within Bahir Dar Zuria, Fogera, and Farta *woredas* that are accessible throughout the course of the year were eligible for inclusion in the *Andilaye* Trial. This decision was made in partnership with relevant *Woreda* Health Offices and One WASH National Program representatives, as officials from these areas were meant to facilitate and supervise the implementation of the *Andilaye* intervention. Officials from relevant *Woreda* Health Offices helped study staff identify *kebeles* that met these eligibility criteria. While sanitation coverage and utilization were originally incorporated as inclusion criteria, the veracity of existing data were not verifiable in many *kebeles* in which initial visits were made. For example, only one latrine was observed in a community in which sanitation coverage was reportedly over 80%, and the community reported this being the case for as long as people could recall. Due to uncertainty regarding sanitation coverage and utilization data, and the challenge of behavioral slippage even in *kebeles* previously declared as open defecation free (ODF), those criteria were dropped from inclusion requirements prior to baseline data collection. As such, any *kebele* meeting the previously stated eligibility criteria was eligible, regardless of sanitation coverage and utilization or previous CLTSH triggering or ODF verification status.

We employed a structured sampling strategy to randomly select eligible *kebeles* within the sampling frame. The primary sampling unit for this study was the *kebele*. The secondary sampling unit for this study was the household; specifically, any household residing in a targeted, sentinel village (*gott*) within a randomly selected study *kebele*. While we randomly selected eligible study clusters (i.e., *kebeles*), we purposively selected *gott(s)*, from which we randomly selected study households. We utilized a ‘fried egg’ [50] approach to purposively select one to two *gotts* that were either situated in or near the center of the *kebele* (if there were centric *gotts*) or were not adjacent to any other study *kebele* (in the event there are no centric *gotts*). This approach minimized spill-over of intervention effects and other externalities associated with the research between intervention and control clusters, especially those adjacent to each other. The number of targeted *gotts* depended only on the number of eligible households identified in *gott* census books. Study household inclusion criteria for the *Andilaye* Trial included any household randomly selected from the *gott* census book residing in the target *gott*(s) that: (1) had at least one child aged 1–9 years at baseline (i.e., the study’s index children) and consented to allowing study staff to observe the children, specifically their faces and hands, and (2) provided consent to participate in the study, with at least one adult household member consenting to serve as the primary survey respondent.

### Recruitment

From the *kebele* sampling frame, we employed a random number generator to identify 50 eligible *kebeles* clusters from across the three *woredas* targeted for our study. Given each of the three *woredas* vary with regard to their hydrogeological conditions and the size and number of *kebeles*, we used a stratified selection approach (at the *woreda* level). Of the 50 clusters, 22 were selected from Farta, 12 from Fogera, and 16 from Bahir Dar Zuria. Proportionally, these selected *kebeles* represented 51 (22/43), 38 (12/32), and 50 (16/32) percent of all *kebeles* in Farta, Fogera, and Bahir Dar Zuria, respectively. An even number of clusters were selected from each *woreda* to ensure an equivalent sample size between the intervention and control clusters selected from each *woreda*. Fogera had a slightly lower proportion of selected *kebeles* due to accessibility concerns given the frequency of floods in the low-land, marshy areas close to Lake Tana (Fig. 1). Our baseline results indicated that 78% (39 of 50) of *kebele* clusters randomly selected for inclusion in the *Andilaye* Trial have been triggered with CLTSH, and certified as ODF according to *Woreda* Health Office records. Another 14% (7 of 50) of study clusters have been triggered with CLTSH but have not yet been certified ODF, and the remaining 8% (4 of 50) have not been triggered with CLTSH, as of the commencement of this study, though they are slated for triggering.

Recruitment took place within the home compounds during baseline data collection. Enumerators contacted adult members of the household; they explained the purpose of the visit, the purpose of the study, and asked the respondent if s/he would be willing to consent to participate in the study using a structured consent form (Additional file 4). Enumerators assessed household level eligibility by asking potential survey respondents a series of questions that lead to a determination of eligibility, and enrolled eligible and consenting households into the trial. During the consent process, enumerators informed respondents that they had the right to choose not to participate in the study, the right to refuse to answer any question, and the right to stop the survey for any reason at any point in time. Enumerators targeted primary survey respondents, based on the following order of priority: (1) the primary female caregiver of the index child, (2) any female household member who serves as a caregiver, (3) any male household member who serves as a caregiver, and (4) any household member over 18 year of age. If households were absent after three visits, no eligible adult respondent was available or refused to consent, or upon further conversation with the household, it became apparent that it was not eligible (e.g., no child aged 1–9 years), the enumerator recorded the information and notified the field supervisor prior to replacing that household with the next randomly selected household on the study roster. Of 1691 surveys initiated during baseline, 1589 (94%) households met all inclusion criteria and were enrolled into the study – 89 households more than the targeted sample size. Targeted households that did not meet inclusion criteria were excluded for the following reasons: 81 households did not have a household member between 1 and 9 years of age, 17 households had no eligible respondent available, three surveys were initiated but not fully completed, and one household did not consent to participate.

### Random allocation

**Fig. 3:**
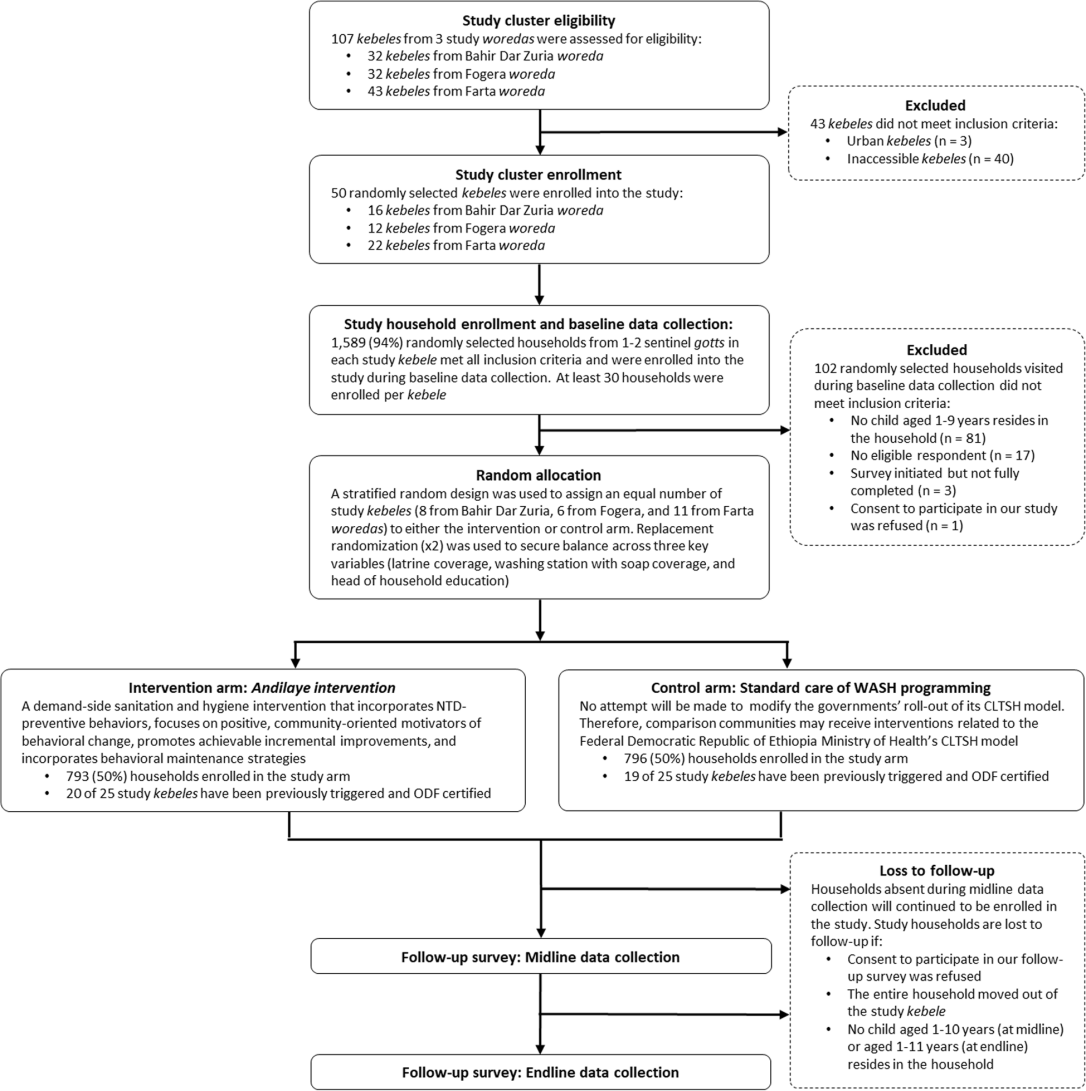
*Andilaye* Trial flow diagram

Following baseline data collection, we used a stratified random design to assign study *kebeles* to either the *Andilaye* intervention or control arm (CLTSH). Within each stratum (*woreda*), researchers from Emory University in Atlanta, USA used a computer-based random number generator to generate a random number between zero and one for each *kebele* cluster, and then placed the clusters in ascending order by their randomly generated numbers. We then partitioned the communities within each *woreda* into two equal sizes, assigning the first half of *kebeles* to the intervention arm and the second half to the control arm. We used replacement re-randomization [51] to secure balance across three key variables (latrine coverage, washing station with soap coverage, and head of household education), as indicated by our baseline survey. CRTs, particularly trials with a small number of clusters, often have individual-level imbalances between study arms. Therefore, we established a priori that the intervention and control mean values for these three variables should be within two standard deviations of the overall mean for these variables. The randomization process described above was repeated (twice) until these variables were balanced according to that *a prior* criterion. Results from our equivalence analyses indicated balance in the number of previously triggered and ODF certified *kebeles*, between study arms, with 20 triggered and ODF *kebeles* in the intervention arm (80%, 20 of 25 study clusters) and 19 in the control arm (76%, 19 of 25 study clusters). Figure 3 provides further details in a flow diagram.

### Implementation

The *Andilaye* intervention is being implemented in collaboration with the *Woreda* Health Offices, HEWs, WDALs, and other community change agents in the 25 *kebeles* randomly selected to the *Andilaye* intervention arm. While allocation occurred at the *kebele* level, intervention activities and data collection occur in one to two sentinel *gotts* per *kebele*, purposively selected to minimize spillover. The counterfactual, or control arm, includes households from *gotts* within randomly selected *kebeles* receiving the FMoH’s standard of care CLTSH programming. As this study is operating in an area where CLTSH is being rolled out nationally, we are not interfering with established CLTSH roll-out and implementation protocols. While we cannot be sure that our control communities will not receive further CLTSH interventions during the course of the trial, any such further implementation of the current CLTSH interventions would only bias effect estimates toward the null.

### Data collection and data management methods

Survey instruments administered at baseline and follow-up data collection rounds (midline and endline) consist of several modules aimed at collecting data on key outcome indicators through reports from respondents and other household members (Additional file 5: Table S3 and Additional file 6: Table S4). When developing these tools, we pulled from a reserve of existing WASH indicators, and leveraged formative research data to contextually adapt survey prompts and answer choices. To the greatest extent possible, we included validated metrics for assessment. Prior to enumerator training, the survey instrument was translated into Amharic, and back-translated by two independent Amharic speakers. The study team discussed and reconciled any discrepancies noted between the intended English prompts and the Amharic translations (identified via the back-translations). In order to ensure face validity, the vast majority of the survey instrument was tested via cognitive interviews. Through the use of this qualitative method, which included ‘think-aloud’ and verbal probing techniques, we obtained feedback from formative research households about the meaning, comprehensiveness, and appropriateness of survey prompts and their related answer choices. Once the Amharic version of the tool was complete, four enumerators were trained on the tool, and administered it during a week-long field pilot in targeted formative research communities. At the end of each day of piloting, the team discussed issues related to respondent comprehension of survey prompts and answer choices, survey logic and skip patterns, and suggested revisions were incorporated, as appropriate. At the end of the piloting period, key data were checked and analyzed, and further modifications were made to the tool prior to finalizing the instrument and supervisor and enumerator trainings. Select finalized survey prompts and answer choices can be found in Additional file 6: Table S4.

The *Andilaye* Trial survey is administered electronically on password-protected mobile phones by enumerators and field supervisors (e.g., completing validation surveys) to improve accuracy of data entry and enable immediate review of results. Data is stored securely using the freely available Open Data Kit (http://opendatakit.org/). Logic, range, and consistency checks were incorporated into the electronic data collection file to further improve data quality and minimize data entry errors. To ensure data quality, the supervisory team, comprised of faculty and staff from Emory University and Emory Ethiopia (a university-derived non-governmental organization in Ethiopia), will coordinate and supervise data collection along with field supervisors. Field supervisors independently assess all objective measures (simultaneous to, but independent of enumerator assessment for validation surveys) at 10% of households in each study *kebele*. Supervisor and enumerator data captured from this sub-set of households will be compared to determine inter-rater reliability of related metrics.

All enumerators and field supervisors are external evaluators who are hired for discrete data collection activities (i.e., baseline, midline, endline, quarterly monitoring). Study staff will not disclose study cluster treatment allocation to field supervisors or enumerators. However, given the nature of the intervention, they may observe intervention materials in *Andilaye* intervention clusters, which may signal treatment allocation.

We will measure and track individual, household, and community-level changes in key outcomes over multiple time points (i.e., baseline, midline, and endline). Households enrolled in the study are lost to follow-up if: (1) consent to participate in our follow-up survey was refused, (2) the entire household moved out of the study *kebele*, or (3) no child aged 1–10 years (at midline) or aged 1–11 years (at endline) resides in the household any longer. Households with no eligible respondent available after three attempts during midline data collection will continue to be enrolled in the study, and will be visited for subsequent quarterly monitoring and endline data collection. A sub-set of key data points will be collected during quarterly monitoring, however, we will seek to minimize reactivity by only engaging half of all households in each cluster per quarter, such that we visit each household for monitoring only once per annum.

### Balance of treatment arms at baseline

Our baseline analyses will establish reference measures for follow-up analyses. We conducted bivariate assessments of balance between study arms at baseline (Table 1). As previously mentioned, 1589 households were enrolled into the study at baseline. Given we prioritized targeting of the primary female caregiver of the index child for baseline survey administration, a large majority (91%) of the respondents were female, by design. Of these 1589 respondents, 85% were the mother of the index child. Demographic variables were balanced across study arms, with no meaningful differences in the prevalence of key demographic variables between arms (Table 1). Similarly, key outcomes of interests were also balanced between the intervention and control arms at baseline. Importantly, facial cleanliness observations of the 1385 index children were similar to all other children aged 1–9 years in the study households (data not shown). This suggests that our index children serve as acceptable sentinels of behavioral outcomes for children of similar ages within the larger household.

At baseline, sanitation and hygiene conditions were found to be generally poor (Table 1). Only 40% of all households had a sanitation facility that was classified as improved based on the WHO/UNICEF Joint Monitoring Programme (JMP) for Water Supply and Sanitation definition. Further, 38% of respondents’ primary place of defecation during the 2 days prior to survey administration was in the open, and only 46% of respondents had defecated in any latrine during the two-day reporting period. These statistics, along with the fact that 39 of 50 *kebele* clusters randomly selected for inclusion in the *Andilaye* Trial have been triggered with CLTSH, and certified ODF, provide strong evidence that behavioral slippage is, indeed, an issue that needs to be addressed in Amhara and perhaps elsewhere in Ethiopia.

Our key health outcome, mental well-being, was shown to be poor, per the WHO-5 scale, amongst approximately one-third of respondents at baseline (Table 1). The baseline prevalence of anxiety, depression, and emotional distress amongst respondents was 29.7, 20.8, and 17.3%, respectively. The distributions of these scores were generally balanced, when comparing the intervention and control arms. Finally, the prevalence of diarrhea during the week (i.e., 7 days) preceding the survey was 9% among index children, and was also generally balanced across study arms (Table 1).

### Analytical methods

The primary method of analysis for all primary and secondary outcomes will follow an “intention-to-treat” analysis, which compares the intervention arm to the control arm, without regard to intervention fidelity or compliance. For binary outcomes, such as our targeted WASH behaviors, we will preferentially use log-linear binomial regression models. For continuous outcomes, such as the mental well-being scale scores, we will use linear regression models. All models will include an intervention variable as a fixed effect, and account for the stratified design through the inclusion of the *woreda* indicator variable [52], and incorporate generalized estimating equations with robust standard errors to account for the clustering of observations within *kebeles*.

The majority of our primary and secondary outcomes (shown in Additional file 5: Table S3) are binary variables, and for these we will preferentially use log-linear binomial models; however, log-linear binomial models are known to have difficulty converging, and so we may instead use modified Poisson regression if we encounter problems with convergence [53]. There is often interest in showing an absolute measure along with a relative measure (e.g., a prevalence ratio), so we will also present prevalence differences. We will use the same models as described above (e.g., log-linear binomial models), but use post-estimation commands to estimate the average marginal effects.

Given no imbalances were detected between study arms at baseline for any of the primary variables of interest (Table 1), we will not need to perform supplementary analyses, as outlined in our pre-analysis plan, to control for the baseline levels of these imbalanced variables in more fully adjusted models. For many of our outcomes, there is interest in determining the impact of the intervention across various sub-groups, such as sex, follow-up round (once multiple rounds of collection are completed), exposure to previous triggering, and modifiers such as water and sanitation insecurity. For select key outcomes, we will use interaction terms and/or stratification, and we will present the impact of the intervention at each level of the sub-group variable (e.g., separately for boys and girls).

Sub-optimal adherence, due perhaps to low uptake of interventions by participants or to poor implementation of the interventions, can lead to trial results that do not reflect the true efficacy of WASH. The intention-to treat analyses show a valid and unbiased causal effect for the effectiveness of a specific WASH program/intervention, but do not show the efficacy of WASH under ideal circumstances. If fidelity and adherence are heterogeneous, we will supplement our primary intention-to-treat analyses with an analysis that attempts to assess the impact of adherence to the *Andilaye* intervention on our primary outcomes of interest (e.g., an instrumental variable analysis, per-protocol analysis, or as-treated analysis). We have performed such analyses in other studies [54, 55].

## Discussion

This study will evaluate the impact of *Andilaye*, a demand-side sanitation and hygiene intervention, on sustained adoption of improved sanitation, personal hygiene, and household environmental sanitation behaviors and mental well-being above and beyond current CLTSH interventions. As outlined below, there are several documented limitations of CLTSH in Ethiopia, and WASH programming more broadly, that the *Andilaye* intervention was designed to address.

1. CLTSH, the key approach to improving sanitation coverage and utilization in Ethiopia, has facilitated considerable changes in coverage of basic sanitation. However, some of these gains have not been sustained, and incremental improvements to improve sanitation (i.e., progress up the sanitation ladder) have not been widely promoted or achieved [29].
2. WASH programs, more broadly, have focused on catalyzing initial behavior change, and have placed little, if any emphasis on the habituation of improved behaviors and behavioral maintenance. Such approaches have fostered behavioral slippage, or regression back to unimproved behaviors and practices, and poor sustainability of behavioral outcomes and potential health impacts [56].
3. CLTSH largely focuses on leveraging shame to change norms around open defecation, but these negative affective motivators may not be the most appropriate or effective drivers of change, and may actually erode mental well-being. As indicated by our formative research, focus on negative affective motivators, poor facilitation of initial triggering, and a lack of post-triggering follow-up has left many communities with negative impressions of CLTSH initiatives.
4. Behaviors and facilities promoted by existing programs are aspirational but require considerable effort and/or capital investment to achieve. A focus on small, incremental improvements in WASH practices and facilities may be viewed as more achievable by program participants, particularly in low resource settings, and as such, may garner greater success.
5. HEWs charged with implementing CLTSH have many responsibilities, few tools, and little capacity to continually reinforce messages [57]. Although Cluster Health Centers are expected to closely support and monitor HEWs, due to a number of reasons, there is limited support extended to them.
6. Siloed approaches within the health and development sectors, namely WASH and those vertical programs involved in the control and elimination of NTDs, prevent the integration and harmonization of NTD and WASH behavior change initiatives [58]. For instance, FMoH’s current CLTSH programming focuses on handwashing with soap at key times, yet overlooks the opportunity of promoting routine facewashing despite the high prevalence of trachoma in the country.
7. While the focus on diarrheal disease prevention and growth faltering have driven investments in WASH, recent evidence suggests that, in sub-Saharan Africa, basic improvements may not be enough to impact these health outcomes [49, 59]. However, factors contributing to the influence of water, sanitation, and hygiene on other important health outcomes, such as mental well-being, remain under-studied.

The *Andilaye* intervention reflects a potential alternative to CLSTH, and evidence generated by the *Andilaye* Trial may serve to address several knowledge gaps related to integrated and holistic community-based, demand-side sanitation and hygiene programming in Ethiopia and beyond. Few community-based WASH interventions have incorporated specific strategies to enhance behavioral maintenance capacity, and limited evidence exists regarding the influence of various inter-personal factors on sustained behavioral change. Evidence generated by this CRT may also indicate how (i.e., through which mechanisms) interventions can bring about sustained improvements in sanitation, hygiene, and less studied health outcomes, such as mental well-being.

Limitations of this CRT include reliance on local government partners to implement and supervise the implementation of the *Andilaye* intervention. Our mixed methods process evaluation will allow us to assess intervention fidelity and adherence, and document any potential bottle-necks that may threaten the intended roll-out of intervention activities. Evidence from the process evaluation will help us interpret the results from the *Andilaye* Trial. Policy-relevant findings and related recommendations regarding the feasibility and effectiveness of the *Andilaye* intervention will be shared broadly when endline data have been collected and analyzed.

## Additional files

Additional file 1: **Figure S1.** Depicts the *Andilaye* logic model. (DOCX 250 kb)

Additional file 2: **Table S1.** Summary of *Andilaye* activities. (DOCX 14 kb)

Additional file 3: **Table S2.** Sample size calculations. (DOCX 13 kb)

Additional file 4: Andilaye Trial consent form. (DOC 65 kb)

Additional file 5: **Table S3.** Key *Andilaye* Trial indicators used to assess behavior and health outcomes. (DOCX 14 kb)

Additional file 6: **Table S4.** Sub-set of *Andilaye* survey prompts and answer choices. (DOCX 51 kb)

## Data Availability

Not applicable. Non-identifiable trial data and statistical code will be made publicly available in a public repository upon the completion of the endline data collection.

## Abbreviations

ARHB: Amhara Regional Health Bureau
CLTSH: community-led total sanitation and hygiene
COM-B: *Capability, Opportunity, Motivation, and Behavior model*
CRT: cluster-randomized trial
DALYs: disability-adjusted life years
FMoH: Federal Ministry of Health
HEP: Health Extension Program
HEWs: Health Extension Workers
HIP: Hygiene Improvement Project
HSCL: Hopkins Symptom Checklist
ICC: intra-class correlation
JMP: WHO/UNICEF Joint Monitoring Programme
NTDs: neglected tropical diseases
ODF: open defecation free
SD: standard deviation
WASH: water, sanitation, and hygiene
WDALs: Women’s Development Army Leaders
WHO: World Health Organization
WSP: Water and Sanitation Program
