# Supplementary figures and images for: Design of a parallel cluster-randomized trial assessing the impact of a demand-side sanitation and hygiene intervention on sustained behavior change and mental well-being in rural and peri-urban Amhara, Ethiopia: Andilaye study protocol
Source: BMC Public Health. 2019 Jun 21;19:801. doi: 10.1186/s12889-019-7040-6 (PMC6588862; doi:10.1186/s12889-019-7040-6)

**Supplemental Figure 1. *Andilaye* logic model**

**
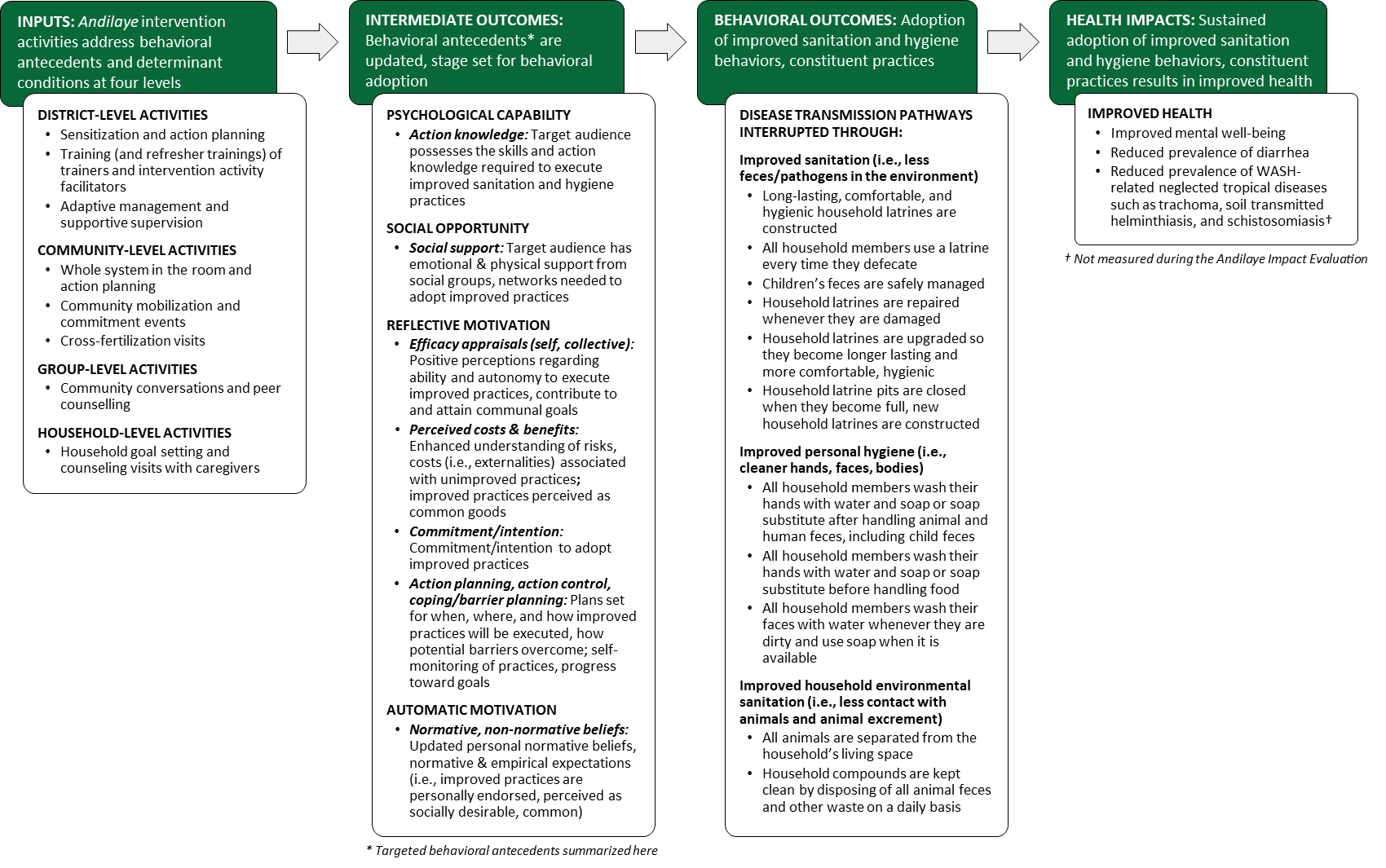
**

Supplement: Supplementary file 1 — Figure S1. Depicts the Andilaye logic model. (DOCX 250 kb) [file 12889_2019_7040_MOESM1_ESM.docx]
