# Supplementary material for: Design of a parallel cluster-randomized trial assessing the impact of a demand-side sanitation and hygiene intervention on sustained behavior change and mental well-being in rural and peri-urban Amhara, Ethiopia: Andilaye study protocol
Source: BMC Public Health. 2019 Jun 21;19:801. doi: 10.1186/s12889-019-7040-6 (PMC6588862; doi:10.1186/s12889-019-7040-6)
Supplement: Supplementary file 2 — Table S1. Summary of Andilaye activities. (DOCX 14 kb) [file 12889_2019_7040_MOESM2_ESM.docx]

**Supplemental Table 1.** **Summary of *Andilaye*  intervention activities – district, community, group, and household levels**

|  | **Activity** | **Aim** |
| --- | --- | --- |
| **District** | Sensitizing and action planning workshop | To orient key stakeholders to the *Andilaye* intervention and engage them in intervention action planning so as to generate buy-in and foster an enabling environment in which the intervention can be implemented. |
|  | Skills-based training of the trainers for HEWs, HEWs Supervisors, *Woreda* officials | To provide skills-based training to HEWs/HEWs Supervisors/*Woreda* officials on household-level intervention activities, supportive supervision, and on-the-job-training so HEWs can, in turn, effectively train WDALs on the implementation of household-level activities and provide supportive supervision. |
|  | Training of community conversation facilitators | To provide comprehensive facilitator training to selected *gott* and *kebele* stakeholders on the ‘community conversations’ group-level intervention activity. |
|  | Skills-based refresher training for supervisors and facilitators | To reinforce previously acquired knowledge and skills and address trainer/facilitator turnover. Prior experience indicates that such trainings serve to sustain actor motivation and further strengthen capacity. |
|  | Adaptive management workshops | To leverage monitoring data to facilitate evidence-based, controlled, and documented operational-specific modifications during critical program moments (i.e., “change gates”). To improve intervention outcomes and resource management by learning from monitored program outcomes. |
| **Community** | Whole system in the room & action planning | To engage key community stakeholders, orient them to the *Andilaye* intervention, and facilitate their involvement in intervention action planning. This participatory approach aims to generate buy-in and foster an enabling environment (i.e., social opportunity) in which the *Andilaye* intervention can be supported and effectively implemented for a “*strong, caring, healthy community*.” |
|  | Skills-based training of WDALs | To provide skills-based training to WDALs on household-level intervention activities, as detailed in the training of the trainers for HEWs, HEWs Supervisors, and *Woreda* officials. |
|  | Skills-based review meetings and refresher trainings for WDALs | To reinforce previously acquired knowledge and skills, address WDAL turnover, and review successes and address challenges faced in implementing counseling visits with caregivers. Prior experience indicates that such trainings serve to sustain actor motivation and further strengthen capacity. |
|  | Community mobilization & commitment event | To improve action knowledge, barrier identification and planning, and attitudes regarding targeted NTD-preventive WASH behaviors through a form of contextually appropriate and interactive edutainment. To initiate the process of shifting social norms through community-generated and managed by-laws and sanctions and public commitment thereof. |
|  | Cross-fertilization visits | To provide an opportunity to share experiences across different intervention communities – to address common implementation bottlenecks, propose solutions, and share perspectives on preliminary behavior change and health outcomes. |
| **Group** | Community conversations | To change factual beliefs and attitudes, enhance action knowledge, improve perceptions of capability, identify and make plans to overcome barriers, and shift social norms regarding targeted behaviors through community group dialogue**.** To carry out demonstrations that address key factors associated with both breaking away from unimproved practices and adopting improved sanitation and hygiene practices. |
|  | Follow-up community conversations | To generate community-level dialogue regarding nuanced issues associated with maintenance of improved practices and barriers thereof through a follow-up round of community group dialog. To carry-out demonstrations related to behavioral maintenance issues. |
| **Household** | Counseling visits with caregivers | To provide personalized counseling to caregivers to equip them with the knowledge, skills, and motivation necessary to adopt improved WASH practices. To foster action capacity, self-efficacy, and barrier planning so caregivers maintain the improved WASH practices. |
|  | Follow-up barrier planning counseling visits with caregivers | To provide continuous follow-up to households such that the house graduates from counseling related to initial adoption of improved practices to counseling related to behavioral maintenance skills. These visits will progressively focus more and more on specific barrier identification and planning skills so the caregiver can maintain his/her improved WASH practices, especially as personal setbacks, systemic shocks, and other obstacles arise. |
