# Supplementary material for: Design of a parallel cluster-randomized trial assessing the impact of a demand-side sanitation and hygiene intervention on sustained behavior change and mental well-being in rural and peri-urban Amhara, Ethiopia: Andilaye study protocol
Source: BMC Public Health. 2019 Jun 21;19:801. doi: 10.1186/s12889-019-7040-6 (PMC6588862; doi:10.1186/s12889-019-7040-6)
Supplement: Supplementary file 3 — Table S2. Sample size calculations. (DOCX 13 kb) [file 12889_2019_7040_MOESM3_ESM.docx]

**Supplemental Table 2.** **Sample size calculations**

|  | **Unexpected prevalence (unexposed)**  **P** | **# in cluster**  **m** | **# of clusters**  **g** | **CI**  **ta .05** | **Error**  **tb .80** | **ICC** | **Magnitude of relative reduction** |
| --- | --- | --- | --- | --- | --- | --- | --- |
| **0.25 baseline** | 0.25 | 20 | 25 | 2 | 0.848 | 0.05 | 44% |
|  | 0.25 | 25 | 25 | 2 | 0.848 | 0.05 | 41% |
|  | 0.25 | 30 | 25 | 2 | 0.848 | 0.05 | 40% |
|  | 0.25 | 35 | 25 | 2 | 0.848 | 0.05 | 39% |
|  | 0.25 | 40 | 25 | 2 | 0.848 | 0.05 | 38% |
| **0.30 baseline** | 0.30 | 20 | 25 | 2 | 0.848 | 0.05 | 38% |
|  | **0.30** | **25** | **25** | **2** | **0.848** | **0.05** | **37%** |
|  | 0.30 | 30 | 25 | 2 | 0.848 | 0.05 | 35% |
|  | 0.30 | 35 | 25 | 2 | 0.848 | 0.05 | 34% |
|  | 0.30 | 40 | 25 | 2 | 0.848 | 0.05 | 33% |
| **0.35 baseline** | 0.35 | 20 | 25 | 2 | 0.848 | 0.05 | 34% |
|  | 0.35 | 25 | 25 | 2 | 0.848 | 0.05 | 33% |
|  | 0.35 | 30 | 25 | 2 | 0.848 | 0.05 | 31% |
|  | 0.35 | 35 | 25 | 2 | 0.848 | 0.05 | 30% |
|  | 0.35 | 40 | 25 | 2 | 0.848 | 0.05 | 30% |
