# Supplementary material for: Design of a parallel cluster-randomized trial assessing the impact of a demand-side sanitation and hygiene intervention on sustained behavior change and mental well-being in rural and peri-urban Amhara, Ethiopia: Andilaye study protocol
Source: BMC Public Health. 2019 Jun 21;19:801. doi: 10.1186/s12889-019-7040-6 (PMC6588862; doi:10.1186/s12889-019-7040-6)
Supplement: Supplementary file 4 — Andilaye Trial consent form. (DOC 65 kb) [file 12889_2019_7040_MOESM4_ESM.doc]

**Oral Consent and Script for The impact of enhanced, demand-side sanitation and hygiene promotion on sustained behavior change and health in Ethiopia**

Household ID: _________________

**Title:** The impact of enhanced, demand-side sanitation and hygiene promotion on sustained behavior change and health in Ethiopia

**Principal Investigator:** Matthew Freeman, PhD, MPH; Rollins School of Public Health, Department of Environmental Health

**Funding Source:** This research is funded by World Bank’s Strategy Impact Evaluation Fund, the International Initiative for Impact Evaluation, and the Children’s Investment Fund Foundation

**Introduction and Study Overview:**

Greetings, my name is ______________. I am working with (indicate Emory Ethiopia or the Ministry of Health). We are visiting your community and other communities in your *woreda* to find out about sanitation, hygiene, water practices, and mental well-being. We want to find out if the sanitation and hygiene measures that have been conducted or will be conducted in your community have any impact on sanitation/hygiene behavior change and/or mental well-being. Your household has been selected to participate in a research study. You can choose whether to participate in the study. Your participation is completely voluntary. I would like to tell you everything you need to think about before you decide whether to participate.

Adults living in all households located in an area that is currently or will soon receive this initiative within this sub-district are eligible to participate. To identify participants, we randomly selected a list of households from your community. All households from this community that are selected to participate in our study will be read an explanation of study procedures, and will be asked for their consent to participate in the study. We randomly selected your household to participate in our study.

A description of this trial will be available on http://www.ClinicalTrials.gov, as required by U.S. law. This website will not include information that can identify you. At most, the website will include a summary of the results. You may search this website at any time if you have access to a computer and internet.

**Procedures:**
If you choose to participate, we will ask you to do two things. First, we will ask you some questions about your household properties; details about your household latrine construction, maintenance, and repairs; the sanitation and hygiene habits of all of your household members; recent health of all of your household members; and your exposure to sanitation and hygiene programme activities. Everything you say will be kept private, and there are no right or wrong answers. Your neighbors and government/non-government partners will never know how you personally respond to the survey. You can refuse to answer any question, and you can end your participation at any time.

After these questions, we will ask you to allow us to observe the faces and hands of your children, and show us all of your household latrines so we can look at and record their structure and condition; and we can measure some distances between the latrine and other important points including your homestead, the latrine water source, and the closest drinking water source to your homestead.

For our observations of your children, we will:

- Look at the faces and hands of all children in the household and make note of their appearance.

This survey, including the interview, direct observation of your latrine, and the examinations should take about one to one and a half hours. We will conduct the survey now and at two additional times in the future, spaced one year apart. A member of our study team will also come back to conduct direct observations about three times per year for the next three years to assess your household latrine and the sanitation and hygiene practices of your household members. That staff member may ask some of the questions we ask you today, but s/he will ask fewer questions.

**Risks and Discomforts:**

There are no known risks from participating in our household interview. You will face little to no risk of physical, psychological, social, or legal injury. Examining your children’s face and hands is simple and painless. While it may be uncomfortable to have our study staff in your home for one to one and a half hours, we are conducting the survey here in order to protect your privacy. We do not expect any harm to come to you or your family because of this survey. There is, however, a possibility that there may be a breach of confidentiality that may result in others becoming knowledgeable of personal information you provide to us during this study. Our team has taken measures to limit the opportunity for breaches in confidentiality. You will not incur any monetary costs for participating in this survey. The only costs you may incur are related to time spent away from your other household activities.

**New Information:**

Certain offices, government agencies, Emory employees, the study donor, and people other than the researchers overseeing proper research protocols may look at study records, but your name and your household’s name will not be provided or linked to any responses you give. These offices include the Emory Institutional Review Board and the Emory Office of Research Compliance. We may also supply our data to other researchers, but our data do not include information that would allow the identities of our study participants to be known. Therefore, if this sharing of data were to take place, it would not result in a breach of confidentiality – your identity will not be known or linked to your survey responses.

**Benefits:**

You may not directly benefit from your participation in this study. You will not be compensated for your participation in this study. However, you and your community may benefit from this study by providing information that may help make sanitation, hygiene, and disease control programming better and may ensure that every household in your community gets these interventions. You will be able to receive benefits and/or assistance from any future programme even if you do not participate in this study.

**Confidentiality:**

Your privacy is very important to us. Information that identifies you and your health status is your “protected health information” (PHI). The PHI for this study includes the GPS coordinate of your household, which we will use to confirm that we are surveying the correct household and calculate the distances between your latrine and other points of interest such as your homestead, latrine water source, and the closest drinking water source. We will also collect information on sanitation and hygiene behaviors and the recent health of you and your household members.

To protect your PHI, we will enter the information into a password-protected computer tablet. Emory Ethiopia will keep any research records we create private to the extent we are required by law. A study number, rather than your name, will be used on study records wherever possible. Your name and other facts that may identify you will not appear when we present or publish study results.

**Voluntary Participation and Withdrawal from the Study:**

Your participation is completely voluntary. There is no penalty if you choose not to participate in the study now, or any time in the future. You have the right to leave the study at any time without penalty. You may refuse to do anything you do not feel comfortable with, or refuse to answer any question you do not wish to answer. If you withdraw from the study, you may request that your responses not be used in our dataset. If you choose to participate in our study, we will keep your answers confidential.

**Contact Information:**

If you have future questions about your rights as a participant or any other questions, concerns, or complaints about the study, please contact Tamiru Kassa Dessalew (Deputy Director of Emory Ethiopia) by phone: 251 923 254 210. If you have future questions about your rights as a research participant or any other questions, concerns, or complaints about the research, contact Endalkachew Deslegn, from the Amhara Regional Health Bureau’s Regional Ethics Committee by telephone at 251 935 466 857.

## Consent:

Do you have any questions about any of the information I just provided? yes/no

Was there anything that seemed unclear? yes/no

If so, I can answer your questions and we can discuss anything that is unclear.

Do you agree to take part in the study? yes/no

Do you agree to let your children take part in the study? yes/no

Enumerator: Record in your tablet whether the individual agrees to participate in the study.

___________________________________________________ _____________________________

Signature of Person Conducting Informed Consent Discussion Date Time
