# Supplementary material for: Design of a parallel cluster-randomized trial assessing the impact of a demand-side sanitation and hygiene intervention on sustained behavior change and mental well-being in rural and peri-urban Amhara, Ethiopia: Andilaye study protocol
Source: BMC Public Health. 2019 Jun 21;19:801. doi: 10.1186/s12889-019-7040-6 (PMC6588862; doi:10.1186/s12889-019-7040-6)
Supplement: Supplementary file 5 — Table S3. Key Andilaye Trial indicators used to assess behavior and health outcomes. (DOCX 14 kb) [file 12889_2019_7040_MOESM5_ESM.docx]

**Supplemental Table 3.** **Key *Andilaye* Trial indicators used to assess behavior and health outcomes**

| **Outcome** | **Indicator group** | **Type of data** |
| --- | --- | --- |
| **WASH behavior change** | Household latrine coverage | - Observation of household compounds - Respondent reported |
|  | Household latrine characteristics | - Observation of household latrines |
|  | Household latrine facility operation and maintenance | - Observation of household latrines - Respondent reported |
|  | Latrine utilization | - Reported (including respondent, head of household, and all household members aged 4-17 years, and waste disposal for children aged 0-3 years) |
|  | Open defecation practices | - Observation of household compounds - Reported (including respondent, head of household, all household members aged 4-17 years) |
|  | Animal husbandry and hygiene practices | - Observation of household compounds - Reported (including respondent, head of household, all household members aged 4-17 years) |
|  | Solid waste management | - Observation of household compounds - Respondent reported |
|  | Face cleaning practices | - Respondent reported (including index child) |
|  | Facial cleanliness | - Observation of index child and other household members aged 1-9 years |
|  | Handwashing practices | - Respondent reported (including index child) |
|  | Hand cleanliness | - Observation of respondent, index child, and other household members aged 1-9 years |
|  | Washing station coverage | - Observation of household compounds - Respondent reported |
| **Diarrhea** | During the last 2 days, three or more loose stools per day | - Respondent reported for index child and other household members aged 1-9 years |
|  | During the last 7 days, three or more loose stools per day | - Respondent reported for index child and other household members aged 1-9 years |
|  | During the last 7 days, blood in the stool | - Respondent reported for index child and other household members aged 1-9 years |
| **Mental well-being, water and sanitation insecurity** | Anxiety symptoms | - Respondent reported |
|  | Depression symptoms | - Respondent reported |
|  | WHO 5 well-being index | - Respondent reported |
|  | Sanitation insecurity | - Respondent reported |
|  | Water insecurity | - Respondent reported |
