# Supplementary material for: Design of a parallel cluster-randomized trial assessing the impact of a demand-side sanitation and hygiene intervention on sustained behavior change and mental well-being in rural and peri-urban Amhara, Ethiopia: Andilaye study protocol
Source: BMC Public Health. 2019 Jun 21;19:801. doi: 10.1186/s12889-019-7040-6 (PMC6588862; doi:10.1186/s12889-019-7040-6)
Supplement: Supplementary file 6 — Table S4. Sub-set of Andilaye survey prompts and answer choices. (DOCX 51 kb) [file 12889_2019_7040_MOESM6_ESM.docx]

**Supplemental Table 4.** **Sub-set of *Andilaye* Trial survey prompts and answer choices**

| **MODULE A: HOUSEHOLD AND RESPONDENT INFORMATION** | | | | | | | | | |
| --- | --- | --- | --- | --- | --- | --- | --- | --- | --- |
| **Question #** | | | | | **Question** | **Response** | | **Constraint** | |
| A.1 | | | | | Woreda name: |  | |  | |
| A.2 | | | | | Kebele name: |  | |  | |
| A.3 | | | | | Gott name: |  | |  | |
| A.4 | | | | | Household number:  *Enumerator note: Refer to the study registry for household number.* | *__ __* | |  | |
| A.5 | | | | | Was consent given by at least one household member who is 18 years or older to participate in this survey and allow the study team to observe all children who are present during the survey?  *Enumerator note: If No, household is not eligible for the survey. Swipe to fill in ‘survey result,’ and then inform field supervisor.* | *Yes 1*  *No 0* | | 🡪 Not eligible for survey | |
| A.6 | | | | | Is at least one household member between the ages of 1-9 years?  *Enumerator note: If No, household is not eligible for the survey. Swipe to fill in ‘survey result,’ and then inform field supervisor.* | *Yes 1*  *No 0* | | 🡪 Not eligible for survey | |
| A.7 | | | | | How many household latrines does your household have access to?  *Enumerator note: All household latrines, including neighbour’s latrines that this household CAN access, should be included in this number.* | *__* | |  | |
| Head of household name | | | | | What is the name of the head of household? |  | |  | |
| A.8 | | | | | What is your (respondent’s) relation to ${head of household name}? | *Not related 0*  *Head of household 1*  *Wife/spouse 2*  *Son/daughter 3*  *Son-in-law/daughter-in-law 4*  *Grandchild 5*  *Parent 6*  *Parent-in-law 7*  *Sibling 8*  *Other relative 9*  *Refused 888*  *Don’t know 999* | |  | |
| A.9 | | | | | What is your (respondent’s) role with regard to household caregiving responsibilities? | *Mother of the index child 1*  *Other female caregiver 2*  *Male caregiver 3*  *Female non-caregiver 4*  *Male non-caregiver 5*  *Refused 888*  *Don’t know 999* | |  | |
| index name | | | | | What is the INDEX child’s name?  *Enumerator note: The index child is defined as the YOUNGEST child in the household who is greater than one year of age, but less than 10 years of age who is present at the home during the survey.* |  | |  | |
| A.10 | | | | | What is the highest education of primary caregiver/mother (index child)? | *No formal school, and illiterate 0*  *No formal schooling, but literate 1*  *Some first cycle primary (grades 1-3) 2*  *Completed first cycle primary (grade 4) 3*  *Some secondary (grades 5-7) 4*  *Completed secondary (grade 8) 5*  *Any high school (grades 9-10) 6*  *Any preparatory (grades 11-12) or above (college/university) 7*  *Refused 888*  *Don’t know 999* | |  | |
| A.11 | | | | | What is the marital status of the primary caregiver/mother (index child)? | *Single 0*  *Married 1*  *Widowed 3*  *Divorced 4*  *Refused 888*  *Don’t know 999* | |  | |
| A.12 | | | | | What is your (respondent’s) age? | *__ __ years* | |  | |
| A.13 | | | | | Indicate respondent’s sex: | *Male 1*  *Female 0* | |  | |
| A.14 | | | | | What is your religion? | *Orthodox Christian 1*  *Muslim 2*  *Protestant 3*  *Catholic 4*  *Traditional belief 5*  *Other 777*  *Refused 888*  *Don’t know 999* | |  | |
| A.15 | | | | | What is your ethnicity? | *Amhara 1*  *Oromo 2*  *Tigre 3*  *Agaw 4*  *Other 777*  *Refused 888*  *Don’t know 999* | |  | |
| A.16 | | | | | During the LAST 7 DAYS, including today, have you gone to fetch water for the household? | *Yes 1*  *No 0*  *Refused 888*  *Don’t know 999* | |  | |
| A.17 | | | | | Do you have any animal herding or other animal husbandry responsibilities (cows, bulls, oxen, goats, sheep, horses, mules, donkeys, chickens)? | *No, none 0*  *Yes, animal herding ONLY (No other animal husbandry responsibilities) 1*  *Yes, animal herding AND other animal husbandry responsibilities 2*  *Yes, other animal husbandry responsibilities BUT NO herding responsibilities 3*  *Refused 888*  *Don’t know 999* | |  | |
| A.18 | | | | | During the LAST 2 DAYS, where was YOUR primary place of defection? | *Household latrine 1*  *Public/community latrine 2*  *Open defecation anywhere INSIDE household compound 3*  *Open defecation anywhere OUTSIDE household compound 4*  *Refused 888*  *Don’t know 999* | |  | |
| A.19 | | | | | Does the primary place of defecation change for YOU over the course of the year? | *Yes 1*  *No 0*  *Refused 888*  *Don’t know 999* | |  | |
| A.20 | | | | | During the LAST 2 DAYS, did YOU openly defecate? | *Yes 1*  *No 0*  *Refused 888*  *Don’t know 999* | | if A.7 !=0 | |
| A.21 | | | | | During the LAST 2 DAYS, did YOU openly defecate in or near a surface water source (e.g. pond, river or lake)? | *Yes 1*  *No 0*  *Refused 888*  *Don’t know 999* | | if A.7 !=0  or A.21 !=0 | |
| A.22 | | | | | During LAST 2 DAYS, did YOU urinate in or near a surface water source (e.g. pond, river or lake)? | *Yes 1*  *No 0*  *Refused 888*  *Don’t know 999* | |  | |
| A.23 | | | | | During the LAST 2 DAYS, did YOU defecate in ANY latrine? | *Yes 1*  *No 0*  *Refused 888*  *Don’t know 999* | |  | |
| A.24 | | | | | During THE LAST 7 DAYS, including today, did YOU ALWAYS EXCLUSIVELY use a latrine for defecation? | *Yes 1*  *No 0*  *Refused 888*  *Don’t know 999* | | if A.7 !=0 | |
| ***Respondent hand cleanliness assessment section*** | | | | | | | | | |
| Note | | | | | OBSERVE respondent’s hand cleanliness via the following criteria: |  | |  | |
| A.25 | | | | | Respondent’s finger nails (Left hand): | *Absence of all visible dirt/mud/debris 0*  *Presence of visible dirt/mud/debris 1* | |  | |
| A.26 | | | | | Respondent’s finger nails (Right hand): | *Absence of all visible dirt/mud/debris 0*  *Presence of visible dirt/mud/debris 1* | |  | |
| A.27 | | | | | Respondent’s length of nails (Left hand): | *Short (i.e., nail at or below finger pad) 1*  *Long (i.e., nail extends beyond finger pad) 2* | |  | |
| A.28 | | | | | Respondent’s length of nails (Right hand): | *Short (i.e., nail at or below finger pad) 1*  *Long (i.e., nail extends beyond finger pad) 2* | |  | |
| A.29 | | | | | Respondent’s finger pads (Left hand): | *Absence of all visible dirt/mud/debris 0*  *Presence of visible dirt/mud/debris 1* | |  | |
| A.30 | | | | | Respondent’s finger pads (Right hand): | *Absence of all visible dirt/mud/debris 0*  *Presence of visible dirt/mud/debris 1* | |  | |
| A.31 | | | | | Respondent’s palms (Left hand): | *Absence of all visible dirt/mud/debris 0*  *Presence of visible dirt/mud/debris 1* | |  | |
| A.32 | | | | | Respondent’s palms (Right hand): | *Absence of all visible dirt/mud/debris 0*  *Presence of visible dirt/mud/debris 1* | |  | |
| ***Household demographic section*** | | | | | | | | | |
| Note | | | | | For this study, household is defined as a person or group of related or unrelated persons who usually live together in the same dwelling unit(s), who have common cooking and eating arrangements, and who acknowledge one adult member as head of household. When we say usually live together, we mean any persons who lives, eats and sleeps in the household 5 or more days per week, and has not been absent from the household for more than 6 months (consecutively) preceding this survey. |  | |  | |
| A.33 | | | | | What is the highest education of ${head of household name}? | *No formal school, and illiterate 0*  *No formal schooling, but literate 1*  *Some first cycle primary (grades 1-3) 2*  *Completed first cycle primary (grade 4) 3*  *Some secondary (grades 5-7) 4*  *Completed secondary (grade 8) 5*  *Any high school (grades 9-10) 6*  *Any preparatory (grades 11-12) or above (college/university) 7*  *Refused 888*  *Don’t know 999* | |  | |
| A.34 | | | | | What is the age of ${head of household name}? | *__ __ years* | |  | |
| A.35 | | | | | What is the sex of ${head of household name}? | *Male 1*  *Female 0* | |  | |
| A.36 | | | | | During the LAST 7 DAYS, including today, has ${head of household name} gone to fetch water for the household? | *Yes 1*  *No 0*  *Refused 888*  *Don’t know 999* | |  | |
| A.37 | | | | | Does ${head of household name} have any animal herding or other animal husbandry responsibilities (cows, bulls, oxen, goats, sheep, horses, mules, donkeys, chickens)? | *No, none 0*  *Yes, animal herding ONLY (No other animal husbandry responsibilities) 1*  *Yes, animal herding AND other animal husbandry responsibilities 2*  *Yes, other animal husbandry responsibilities BUT NO herding responsibilities 3*  *Refused 888*  *Don’t know 999* | |  | |
| A.38 | | | | | If present, OBSERVE whether ${head of household name} is wearing shoes? | *Head of household is NOT present for observation 666*  *Head of household is not wearing shoes 0*  *Head of household is wearing shoes 1* | |  | |
| A.39 | | | | | During the LAST 2 DAYS, where was ${head of household name}’s primary place of defecation? | *Household latrine 1*  *Public/community latrine 2*  *Open defecation anywhere INSIDE household compound 3*  *Open defecation anywhere OUTSIDE household compound 4*  *Refused 888*  *Don’t know 999* | |  | |
| A.40 | | | | | Does the primary place of defecation change for ${head of household name} over the course of the year? | *Yes 1*  *No 0*  *Refused 888*  *Don’t know 999* | |  | |
| A.41 | | | | | During the LAST 2 DAYS, did ${head of household name} openly defecate? | *Yes 1*  *No 0*  *Refused 888*  *Don’t know 999* | | if A.7 !=0 | |
| A.42 | | | | | During the LAST 2 DAYS, did ${head of household name} openly defecate in or near a surface water source (e.g. pond, river, or lake)? | *Yes 1*  *No 0*  *Refused 888*  *Don’t know 999* | | if A.7 !=0  or A.41 !=0 | |
| A.43 | | | | | During the LAST 2 DAYS, did ${head of household name} urinate in or near a surface water source (e.g. pond, river, or lake)? | *Yes 1*  *No 0*  *Refused 888*  *Don’t know 999* | |  | |
| A.44 | | | | | During the LAST 2 DAYS, did ${head of household name} defecate in ANY latrine? | *Yes 1*  *No 0*  *Refused 888*  *Don’t know 999* | |  | |
| A.45 | | | | | During the LAST 7 DAYS, including today, did ${head of household name} ALWAYS EXCLUSIVELY use a latrine for defecation? | *Yes 1*  *No 0*  *Refused 888*  *Don’t know 999* | | if A.7 !=0 | |
| A.46 | | | | | Enter the type of report for ${head of household name}’s information: | *Self-report 1*  *Proxy report (i.e., report made on behalf of the person) 2*  *Refused 888*  *Don’t know 999* | |  | |
| A.47 | | | | | How many children between 0 and 9 years of age live in the household?  *Enumerator note: Enter 999 if respondent doesn’t know or can’t recall. Enter 888 if respondent refused to answer.* | *__ __ __ children* | |  | |
| A.48 | | | | | How many people 10 to 17 years of age live in the household?  *Enumerator note: Enter 999 if respondent doesn’t know or can’t recall. Enter 888 if respondent refused to answer* | *__ __ __ people* | |  | |
| A.49 | | | | | How many people 18 years or older live in the household?  *Enumerator note: Enter 999 if respondent doesn’t know or can’t recall. Enter 888 if respondent refused to answer* | *__ __ __ people* | |  | |
| ***Household member census*** | | | | | | | | | |
| Note | | | | | When we say “household member,” we mean any persons who lives, eats and sleeps in the household 5 or more days per week, and has not been absent from the household for more than 6 months (consecutively) preceding this survey. |  | |  | |
| Note | | | | | READ OUT LOUD TO THE RESPONDENT: "We would like to know information about each person aged 0 to 17 years, who usually lives in your household. We will collect information on all household members in this age range, starting with the youngest person in your household, and moving to the next youngest and onward until we collect information on ALL members of the household aged 0 to 17 years. Even if this person is not physically present at the household at the moment, we would like to ask you some questions about him/her so we have information on all household members." |  | |  | |
| member name | | | | | Household member name: |  | | *Repeat this section for each household member* | |
| A.47 | | | | | What is ${member name}’s age (in years)?  *Enumerator note: If child is < 1 year, enter 0. If respondent doesn't know or can't recall, enter your estimate of the age. Enter 888 if respondent refused to answer.* | *__ __ __ years* | |  | |
| A.48 | | | | | Indicate whether ${member name} is the index child (${index name}): | *Yes 1*  *No 0* | | if 0>A.47<10 | |
| A.49 | | | | | Indicate ${member name}’s sex: | *Male 1*  *Female 0* | |  | |
| A.50 | | | | | Is ${member name} present at the household at the moment (i.e., during the survey)? | *Absent from house (e.g., at the market, traveling, working away from the house) 0*  *Present during survey 1*  *At school 2* | |  | |
| A.51 | | | | | Is ${member name} currently enrolled in school? | *Yes 1*  *No 0* | | if 4>A.47<18 | |
| A.52 | | | | | During the LAST 7 DAYS, including today, has ${member name} gone to fetch water for the household? | *Yes 1*  *No 0*  *Refused 888*  *Don’t know 999* | | if 3>A.47<18 | |
| A.53 | | | | | Does ${member name} have any animal herding or other animal husbandry responsibilities (cows, bulls, oxen, goats, sheep, horses, mules, donkeys, chickens)? | *No, none 0*  *Yes, animal herding ONLY (No other animal husbandry responsibilities) 1*  *Yes, animal herding AND other animal husbandry responsibilities 2*  *Yes, other animal husbandry responsibilities BUT NO herding responsibilities 3*  *Refused 888*  *Don’t know 999* | | if 3>A.47<18 | |
| A.54 | | | | | OBSERVE: whether ${member name} is currently wearing shoes | *Yes 1*  *No 0* | | if A.50=1 | |
| A.55 | | | | | During the LAST 2 DAYS, has ${member name} had nasal congestion? | *Yes 1*  *No 0*  *Refused 888*  *Don’t know 999* | | if -1<A.47<10 | |
| A.56 | | | | | During the LAST 2 DAYS, has ${member name} run a fever? | *Yes 1*  *No 0*  *Refused 888*  *Don’t know 999* | |  | |
| A.57 | | | | | In the LAST 2 DAYS, has ${member name} had three or more loose stools per day (diarrhoea)? | *Yes 1*  *No 0*  *Refused 888*  *Don’t know 999* | |  | |
| A.58 | | | | | During the LAST 7 DAYS, including today, has ${member name} had three or more loose stools per day (diarrhoea)? | *Yes 1*  *No 0*  *Refused 888*  *Don’t know 999* | | if A.57 !=1 | |
| A.59 | | | | | During the LAST 7 DAYS, including today, has ${member name} had blood in the stool (dysentery)? | *Yes 1*  *No 0*  *Refused 888*  *Don’t know 999* | |  | |
| A.60 | | | | | During the LAST 7 DAYS, including today, has ${member name} been involved in any accidents? | *Yes 1*  *No 0*  *Refused 888*  *Don’t know 999* | |  | |
| A.61 | | | | | During the LAST 7 DAYS, including today, has ${member name} experienced heartburn? | *Yes 1*  *No 0*  *Refused 888*  *Don’t know 999* | |  | |
| ***Latrine utilization section*** | | | | | | | | | |
| Note | | | | | READ OUT LOUD TO THE RESPONDENT: “We will now ask about household latrine use.  If ${member name} is present and over the age of 10 years, please have ${member name} respond for himself/herself.” |  | |  | |
| A.62 | | | | | During the LAST 2 DAYS, where was ${member name}’s primary place of defecation? | *Household latrine 1*  *Public/community latrine 2*  *Open defecation anywhere INSIDE household compound 3*  *Open defecation anywhere OUTSIDE household compound 4*  *Refused 888*  *Don’t know 999* | |  | |
| A.63 | | | | | Does the primary place of defecation change for ${member name} over the course of the year? | *Yes 1*  *No 0*  *Refused 888*  *Don’t know 999* | |  | |
| A.64 | | | | | During the LAST 2 DAYS, did ${member name} openly defecate? | *Yes 1*  *No 0*  *Refused 888*  *Don’t know 999* | |  | |
| A.65 | | | | | During the LAST 2 DAYS, did ${member name} openly defecate in or near a surface water source (e.g. pond, river, or lake)? | *Yes 1*  *No 0*  *Refused 888*  *Don’t know 999* | | if A.64 !=0 | |
| A.66 | | | | | During the LAST 2 DAYS, did ${member name} urinate in or near a surface water source (e.g. pond, river, or lake)? | *Yes 1*  *No 0*  *Refused 888*  *Don’t know 999* | |  | |
| A.67 | | | | | During the LAST 2 DAYS, did ${member name} defecate in ANY latrine? | *Yes 1*  *No 0*  *Refused 888*  *Don’t know 999* | |  | |
| A.68 | | | | | During the LAST 7 DAYS, including today, did ${member name} ALWAYS EXCLUSIVELY use a latrine for defecation? | *Yes 1*  *No 0*  *Refused 888*  *Don’t know 999* | |  | |
| A.69 | | | | | Enumerator: Enter the type of report for ${member name}’s information: | *Self-report 1*  *Proxy report (i.e., report made on behalf of the person) 2*  *Refused 888*  *Don’t know 999* | |  | |
| A.70 | | | | | The last time ${member name} passed stools, what happened to the stools? | *Child used the latrine 666*  *Nothing, the feces were left in the open 0*  *The faeces were put/rinsed into a surface water source, drain, ditch, or the bush/yard 1*  *The faeces were thrown into the garbage 2*  *The faeces were buried 3*  *The faeces were put/rinsed into the latrine 4*  *Other 777* | | if -1<A.47<4 | |
| ***Hand cleanliness assessment section*** | | | | | | | | | |
| Note | | | | | OBSERVE ${member name}’s hand cleanliness via the following criteria: |  | |  | |
| A.71 | | | | | ${member name}’s finger nails (Left hand): | *Absence of all visible dirt/mud/debris 0*  *Presence of visible dirt/mud/debris 1* | |  | |
| A.72 | | | | | ${member name}’s finger nails (Right hand): | *Absence of all visible dirt/mud/debris 0*  *Presence of visible dirt/mud/debris 1* | |  | |
| A.73 | | | | | ${member name}’s length of nails (Left hand): | *Short (i.e., nail at or below finger pad) 1*  *Long (i.e., nail extends beyond finger pad) 2* | |  | |
| A.74 | | | | | ${member name}’s length of nails (Right hand): | *Short (i.e., nail at or below finger pad) 1*  *Long (i.e., nail extends beyond finger pad) 2* | |  | |
| A.75 | | | | | ${member name}’s finger pads (Left hand): | *Absence of all visible dirt/mud/debris 0*  *Presence of visible dirt/mud/debris 1* | |  | |
| A.76 | | | | | ${member name}’s finger pads (Right hand): | *Absence of all visible dirt/mud/debris 0*  *Presence of visible dirt/mud/debris 1* | |  | |
| A.77 | | | | | ${member name}’s palms (Left hand): | *Absence of all visible dirt/mud/debris 0*  *Presence of visible dirt/mud/debris 1* | |  | |
| A.78 | | | | | ${member name}’s palms (Right hand): | *Absence of all visible dirt/mud/debris 0*  *Presence of visible dirt/mud/debris 1* | |  | |
| ***Facial cleanliness assessment section*** | | | | | | | | | |
| note | | | | | OBSERVE ${member name}’s facial cleanliness using the following criteria: |  | |  | |
| A.79 | | | | | OBSERVE whether ocular discharge is absent or present on ${member name}’s face | *Absent 0*  *Present 1* | |  | |
| A.80 | | | | | OBSERVE whether WET nasal discharge is absent or present on ${member name}’s face | *Absent 0*  *Present 1* | |  | |
| A.81 | | | | | OBSERVE whether DRY nasal discharge is absent or present on ${member name}’s face | *Absent 0*  *Present 1* | |  | |
| A.81 | | | | | OBSERVE whether other dirt/dust/other debris is absent or present on ${member name}’s face | *Absent 0*  *Present 1* | |  | |
| A.82 | | | | | OBSERVE ${index name}’s face for 1 minute. Count the number of times a fly lands on the child’s face.  Enter the number of times you observed a fly land on ${index name}’s face during the 1 minute of observation. | *__ __ times* | | if A.48=1 | |
| **MODULE B: WATER MODULE** | | | | | | | | | |
| **Question #** | | | | **Question** | | | **Response** | | **Constraint** |
| B.1 | | | | READ THE FOLLOWING OUT LOUD TO THE RESPONDENT: “Please show me all of the containers you/whoever fetches water. How many of these containers do you/they use per trip to COLLECT water?”  Number of containers used to collect/fetch water: | | | *__ __ containers* | |  |
| B.2 | | | | Estimated volume of all containers used to collect/fetch water, in liters: | | | *__ __ __ __ liters* | | if B.1 !=0 |
| B.3 | | | | Number of small-necked containers used to fetch water: | | | *__ __ small-necked containers* | | if B.1 !=0 |
| B.4 | | | | READ THE FOLLOWING OUT LOUD TO THE RESPONDENT: “Please show me all of the containers your household uses to STORE water.”  Number of containers: | | | *__ __ containers* | |  |
| B.5 | | | | Number of small-necked containers used to store water: | | | *__ __ small-necked containers* | | if B.1 !=0 |
| B.6 | | | | Did you do anything to treat THIS water? | | | *Yes 1*  *No 0*  *Refused 888*  *Don’t know 999* | |  |
| B.7 | | | | What method of treatment did you use to treat THIS water? | | | *Boiled the water 1*  *Filtered the water 2*  *Chlorinated the water 3*  *Other treatment 777*  *Refused 888*  *Don’t know 999* | | if B.6=1 |
| B.8 | | | | During the LAST 7 DAYS, including today, on how many days did a member of your household fetch water? | | | *Zero 0*  *One 1*  *Two 2*  *Three 3*  *Four 4*  *Five 5*  *Six 6*  *Seven 7* | |  |
| B.9 | | | | On the LAST DAY your household collected water, how many trips did members of your household make to the water source?  *Enumerator note: If two people went one time each, that counts as two trips* | | | *__ __ trips* | |  |
| B.10 | | | | What is the main source of DRINKING WATER for members of your household? | | | *Surface water 13*  *Unprotected dug well 6*  *Unprotected spring 8*  *Public tap/standpost 3*  *Tubewell/borehole 4*  *Protected dug well 5*  *Protected spring 7*  *Rainwater collection 9*  *Piped water into dwelling 1*  *Piped water into yard/plot 2*  *Bottled water 10*  *Cart with small tank/drum 11*  *Tanker-truck 12*  *Refused 888*  *Don’t know 999* | |  |
| B.11 | | | | How many months is this main source of DRINKING WATER available for use? | | | *__ __ months* | | *This number must be between 1 and 12 months* |
| B.12 | | | | Is this main source of DRINKING WATER located within your own household compound? | | | *Yes 1*  *No 0*  *Refused 888*  *Don’t know 999* | |  |
| B.13 | | | | Currently, how long does it take to go to the main source of DRINKING WATER, get water, & come back (in min)? | | | *__ __ __ minutes* | | if B.12 !=1 |
| B.13_hours | | | |  | | |  | | Calculate hours for B.13 |
| B.14 | | | | Enumerator, verify the time with the participant in minutes and hours by saying “${B.13} minutes is ${B.13_hours} hours. So it takes about that long to go to the main source of DRINKING WATER, get water & come back?” | | |  | | if B.12 !=1 |
| B.15 | | | | What is the main source of water used by your household for other purposes, such as cooking and handwashing? | | | *Same as main drinking water source 0*  *Surface water 13*  *Unprotected dug well 6*  *Unprotected spring 8*  *Public tap/standpost 3*  *Tubewell/borehole 4*  *Protected dug well 5*  *Protected spring 7*  *Rainwater collection 9*  *Piped water into dwelling 1*  *Piped water into yard/plot 2*  *Bottled water 10*  *Cart with small tank/drum 11*  *Tanker-truck 12*  *Refused 888*  *Don’t know 999* | |  |
| B.16 | | | | Is this main source of water used for other purposes, such as cooking and handwashing located within your own household compound? | | | *Yes 1*  *No 0*  *Refused 888*  *Don’t know 999* | | if B.15 !=0 |
| B.17 | | | | Currently, how long does it take to go to the source used for other purposes, get water, & come back (in min)?  *This time should be entered in MINUTES* | | | *__ __ __ minutes* | | if B.15 !=0  or B.16 =1 |
| B.17_hours | | | |  | | |  | | Calculate hours for B.13 |
| B.18 | | | | Enumerator, verify the time with the participant in minutes and hours by saying: “${B.17} minutes is ${B.17_hours} hours. So it takes about that long to go to the source used for other purposes, get water & come back?” | | |  | | if B.16 !=1 |
| ***Water insecurity scale*** | | | | | | | | | |
| Note | | | | READ THE FOLLOWING OUT LOUD TO THE RESPONDENT: “Now I’m going to ask you some questions, and I would like you to provide answers based on your experience in the LAST 3 months.” | | |  | |  |
| B.19 | | | | In the past 3 months, did you worry that you would not have enough water for all of your household needs? This includes water for cooking, bathing, cleaning as well as for animals, crops, and businesses. | | | *Yes 1*  *No 0* | |  |
| B.20 | | | | Was that just a few times or often? | | | *A few 1*  *Often 2* | | if B.19=1 |
| B.21 | | | | In the past 3 months, did you ever do less cleaning, drinking, cooking, or personal bathing because your household did not have enough water? | | | *Yes 1*  *No 0* | |  |
| B.22 | | | | Was that just a few times or often? | | | *A few 1*  *Often 2* | | if B.21=1 |
| B.23 | | | | In the past 3 months, did you or anyone in your household drink water that you thought/worried might not be safe for health? | | | *Yes 1*  *No 0* | |  |
| B.24 | | | | Was that just a few times or often? | | | *A few 1*  *Often 2* | | if B.23=1 |
| B.25 | | | | In the past 3 months, did you or someone in your household not cook a desirable food because there was not enough water? | | | *Yes 1*  *No 0* | |  |
| B.26 | | | | Was that just a few times or often? | | | *A few 1*  *Often 2* | | if B.25=1 |
| B.27 | | | | In the past 3 months, did you or anyone else in your household go to sleep thirsty because there was not enough water? | | | *Yes 1*  *No 0* | |  |
| B.28 | | | | Was that just a few times or often? | | | *A few 1*  *Often 2* | | if B.27=1 |
| B.29 | | | | In the past 3 months, did you or any household member go to sleep hungry at night because there was not enough food? | | | *Yes 1*  *No 0* | |  |
| B.30 | | | | Was that just a few times or often? | | | *A few 1*  *Often 2* | | if B.29=1 |
| **MODULE C: HYGIENE MODULE** | | | | | | | | | |
| **Question #** | **Question** | | | | | | **Response** | | **Constraint** |
| ***Handwashing*** | | | | | | | | | |
| Note | Ask the following questions, but DO NOT read out the answer choices. Listen to the response, and enter the appropriate answer choice. | | | | | |  | |  |
| C.1 | Yesterday, did ${index name}’s HANDS get washed (by you, the child, or anyone else? | | | | | | *Yes 1*  *No 0*  *Refused 888*  *Don’t know 999* | |  |
| C.2 | The LAST time ${index name}’s hands got washed, was soap/ash/or soapy water used? | | | | | | *Yes 1*  *No 0*  *Refused 888*  *Don’t know 999* | | if C.1 !=0 |
| C.3 | Yesterday, did YOU wash YOUR hands with water? | | | | | | *Yes 1*  *No 0*  *Refused 888*  *Don’t know 999* | |  |
| C.4 | The LAST time you washed your hands, did you use soap/ash/or soapy water? | | | | | | *Yes 1*  *No 0*  *Refused 888*  *Don’t know 999* | |  |
| C.5 | Yesterday, was surface water (e.g., pond, river, or lake) used for handwashing?  *Enumerator note: We are interested in knowing whether the index child OR respondent used surface water for handwashing.* | | | | | | *Yes 1*  *No 0*  *Refused 888*  *Don’t know 999* | | if C.1 !=0  or C.3 !=0 |
| C.6 | Yesterday, did you have sufficient water for handwashing? | | | | | | *Yes 1*  *No 0*  *Refused 888*  *Don’t know 999* | |  |
| C.7 | The last time ${index name} defecated, did s/he clean his/her hands with water and soap, soapy water or ash? | | | | | | *Yes 1*  *No 0*  *Refused 888*  *Don’t know 999* | |  |
| C.8 | The last time you defecated, did you clean your hands with water and soap, soapy water, or ash? | | | | | | *Yes 1*  *No 0*  *Refused 888*  *Don’t know 999* | |  |
| C.9 | The last time you prepared food, did you clean your hands with water and soap, soapy water, or ash before beginning the food preparations? | | | | | | *Yes 1*  *No 0*  *Refused 888*  *Don’t know 999* | |  |
| ***Facewashing*** | | | | | | | | | |
| C.10 | Yesterday, did ${index name}’s FACE get cleaned by you, the child, or anyone else? | | | | | | *Yes 1*  *No 0*  *Refused 888*  *Don’t know 999* | |  |
| C.11 | Yesterday, how did ${index_name}’s FACE get cleaned? | | | | | | *By wiping the eyes with hands or a cloth 0*  *With saliva 1*  *With water only 2*  *With water and soap or soapy water 3*  *Other technique 777* | | if C.10=1 |
| C.12 | Yesterday, after ${index_name}’s FACE was washed, was it wiped dry with a cloth such as a towel or apron? | | | | | | *Yes 1*  *No 0*  *Refused 888*  *Don’t know 999* | | if C.11=2  or C.11=3 |
| C.13 | Yesterday, did YOU clean YOUR face? | | | | | | *Yes 1*  *No 0*  *Refused 888*  *Don’t know 999* | |  |
| C.14 | Yesterday, how did you clean your face? | | | | | | *By wiping the eyes with hands or a cloth 0*  *With saliva 1*  *With water only 2*  *With water and soap or soapy water 3*  *Other technique 777* | | if C.13=1 |
| C.15 | Yesterday, after you washed YOUR FACE, did you wipe it dry with a cloth such as a towel or apron? | | | | | | *Yes 1*  *No 0*  *Refused 888*  *Don’t know 999* | | if C.14=2  or C.14=3 |
| C.16 | Yesterday, was surface water (e.g., pond,river or lake) used for facewashing?  *Enumerator note: We are interested in knowing whether the index child OR respondent used surface water for facewashing.* | | | | | | *Yes 1*  *No 0*  *Refused 888*  *Don’t know 999* | | **i**f C.11=2  or C.11=3  or C.14=2  or C.14=3 |
| C.17 | Yesterday did you have sufficient water for facewashing? | | | | | | *Yes 1*  *No 0*  *Refused 888*  *Don’t know 999* | |  |
| ***Other personal hygiene*** | | | | | | | | | |
| C.18 | During the last 7 days, including today, how many times did ${index_name} bathe or get bathed by another? | | | | | | *Never in the last 7 days 0*  *One time in the last 7 days 1*  *Two times in the last 7 days (every third day) 2*  *Three to six times in the last 7 days (every other day to most days) 3*  *Once per day (7 of the last 7 days) 7*  *More than once per day (more than 7 times in the last 7 days) 8*  *N/A – don’t own the item 666*  *Refused 888*  *Don’t know 999* | |  |
| C.19 | During the last 7 days, including today, how many times did YOU bathe your body? | | | | | | *Never in the last 7 days 0*  *One time in the last 7 days 1*  *Two times in the last 7 days (every third day) 2*  *Three to six times in the last 7 days (every other day to most days) 3*  *Once per day (7 of the last 7 days) 7*  *More than once per day (more than 7 times in the last 7 days) 8*  *N/A – don’t own the item 666*  *Refused 888*  *Don’t know 999* | |  |
| C.20 | During the last 7 days, including today, did you have sufficient water for bathing? | | | | | | *Yes 1*  *No 0*  *Refused 888*  *Don’t know 999* | |  |
| C.21 | During the last 7 days, including today, was surface water (e.g., pond,river or lake) used for bathing?  *We are interested in knowing whether the index child OR respondent used surface water for bathing.* | | | | | | *Yes 1*  *No 0*  *Refused 888*  *Don’t know 999* | | if C.18 !=0  or C.19 !=0 |
| Note | During the last 7 days, including today, how many times did you wash the following items: | | | | | |  | |  |
| C.22 | ${index name}’s clothes | | | | | | *Never in the last 7 days 0*  *One time in the last 7 days 1*  *Two times in the last 7 days (every third day) 2*  *Three to six times in the last 7 days (every other day to most days) 3*  *Once per day (7 of the last 7 days) 7*  *More than once per day (more than 7 times in the last 7 days) 8*  *N/A – don’t own the item 666*  *Refused 888*  *Don’t know 999* | |  |
| C.23 | YOUR own clothes | | | | | | *Never in the last 7 days 0*  *One time in the last 7 days 1*  *Two times in the last 7 days (every third day) 2*  *Three to six times in the last 7 days (every other day to most days) 3*  *Once per day (7 of the last 7 days) 7*  *More than once per day (more than 7 times in the last 7 days) 8*  *N/A – don’t own the item 666*  *Refused 888*  *Don’t know 999* | |  |
| C.24 | Towels | | | | | | *Never in the last 7 days 0*  *One time in the last 7 days 1*  *Two times in the last 7 days (every third day) 2*  *Three to six times in the last 7 days (every other day to most days) 3*  *Once per day (7 of the last 7 days) 7*  *More than once per day (more than 7 times in the last 7 days) 8*  *N/A – don’t own the item 666*  *Refused 888*  *Don’t know 999* | |  |
| C.25 | Bedsheets | | | | | | *Never in the last 7 days 0*  *One time in the last 7 days 1*  *Two times in the last 7 days (every third day) 2*  *Three to six times in the last 7 days (every other day to most days) 3*  *Once per day (7 of the last 7 days) 7*  *More than once per day (more than 7 times in the last 7 days) 8*  *N/A – don’t own the item 666*  *Refused 888*  *Don’t know 999* | |  |
| C.26 | Baby carrier | | | | | | *Never in the last 7 days 0*  *One time in the last 7 days 1*  *Two times in the last 7 days (every third day) 2*  *Three to six times in the last 7 days (every other day to most days) 3*  *Once per day (7 of the last 7 days) 7*  *More than once per day (more than 7 times in the last 7 days) 8*  *N/A – don’t own the item 666*  *Refused 888*  *Don’t know 999* | |  |
| C.27 | Children’s toys | | | | | | *Never in the last 7 days 0*  *One time in the last 7 days 1*  *Two times in the last 7 days (every third day) 2*  *Three to six times in the last 7 days (every other day to most days) 3*  *Once per day (7 of the last 7 days) 7*  *More than once per day (more than 7 times in the last 7 days) 8*  *N/A – don’t own the item 666*  *Refused 888*  *Don’t know 999* | |  |
| C.28 | During the last 7 days, including today, did you have sufficient water for washing clothes? | | | | | | *Yes 1*  *No 0*  *Refused 888*  *Don’t know 999* | |  |
| C.29 | During the last 7 days, including today, was surface water (e.g., pond, river, or lake) used for washing clothes?  *Enumerator note: We are interested in knowing whether the respondent used surface water for washing items.* | | | | | | *Yes 1*  *No 0*  *Refused 888*  *Don’t know 999* | | if C.22 !=0  or C.23 !=0  or C.25 !=0  or C.26 !=0  or C.27 !=0  or C.28 !=0 |
| **MODULE D: HOUSEHOLD ASSETS & WEALTH INDICATOR MODULE** | | | | | | | | | |
| **Question #** | | **Question** | | | | | **Response** | | **Constraint** |
| Note | | Read through each asset, and use the code below to mark whether the household owns the asset | | | | |  | |  |
| D.1 | | In your household is there: | | | | | *Yes 1*  *No 0*  *Refused 888*  *Don’t know 999* | |  |
| D.2 | | Working electricity | | | | | *Yes 1*  *No 0*  *Refused 888*  *Don’t know 999* | |  |
| D.3 | | Functioning radio set | | | | | *Yes 1*  *No 0*  *Refused 888*  *Don’t know 999* | |  |
| D.4 | | Functioning television | | | | | *Yes 1*  *No 0*  *Refused 888*  *Don’t know 999* | |  |
| D.5 | | Functioning bicycle | | | | | *Yes 1*  *No 0*  *Refused 888*  *Don’t know 999* | |  |
| D.6 | | Functioning motorcycle/scooter | | | | | *Yes 1*  *No 0*  *Refused 888*  *Don’t know 999* | |  |
| D.7 | | Functioning car/truck | | | | | *Yes 1*  *No 0*  *Refused 888*  *Don’t know 999* | |  |
| D.8 | | Functioning mobile phone | | | | | *Yes 1*  *No 0*  *Refused 888*  *Don’t know 999* | |  |
| D.9 | | Functioning mitad | | | | | *Yes 1*  *No 0*  *Refused 888*  *Don’t know 999* | |  |
| D.10 | | Functioning kerosene or pressure lamp | | | | | *Yes 1*  *No 0*  *Refused 888*  *Don’t know 999* | |  |
| D.11 | | One or more beds and one or more tables | | | | | *Yes 1*  *No 0*  *Refused 888*  *Don’t know 999* | |  |
| D.12 | | An enclosed bathing area | | | | | *Yes 1*  *No 0*  *Refused 888*  *Don’t know 999* | |  |
| D.13 | | Number of cattle owned by household (cows/bulls/oxen) | | | | | *__ __ __ cattle* | |  |
| D.14 | | Number of horses, mules, or donkeys owned by household | | | | | *__ __ __ horses, mules or donkeys* | |  |
| D.15 | | Number of sheep or goats owned by household | | | | | *__ __ __ sheep or goats* | |  |
| D.16 | | Number of chickens owned by household | | | | | *__ __ __ chickens* | |  |
| Note | | Do any of the members of your household own the following: | | | | |  | |  |
| D.17 | | A homestead/house | | | | | *Yes 1*  *No 0*  *Refused 888*  *Don’t know 999* | |  |
| D.18 | | Crop land | | | | | *Yes 1*  *No 0*  *Refused 888*  *Don’t know 999* | |  |
| D.19 | | Cash crops  *Enumerator note: A cash crop is a crop that is grown primarily for selling. While a household may use some of the crop for subsistence (feeding family members and animals), that is not the main reason the household is growing the crop.* | | | | | *Yes 1*  *No 0*  *Refused 888*  *Don’t know 999* | |  |
| D.20 | | Do any of the members of your household work their own or their family’s agricultural land? | | | | | *Yes 1*  *No 0*  *Refused 888*  *Don’t know 999* | |  |
| D.21 | | What is the principal type of fuel for cooking used by your household? | | | | | *Electricity 1*  *Gas 2*  *Kerosene 3*  *Charcoal 4*  *Wood 5*  *Dung, manure 6*  *Corn cobbs or other biofuel 7*  *Other 777*  *Refused 888*  *Don’t know 999* | |  |
| D.22 | | What was the main source of cash income of all family members in the last one year? | | | | | *Selling crops 1*  *Selling animal products 2*  *Fishing 3*  *Daily labour 4*  *Farm labour 5*  *Business/trade 6*  *Salary 7*  *Other (e.g., gifts from others) 777*  *Refused 888*  *Don’t know 999* | |  |
| D.23 | | How well off do you think your household is in relation to other households in this village?  *Enumerator note: READ the response options* | | | | | *Better off than other households 1*  *The same as other households 2*  *Worse off than other households 3*  *Refused 888*  *Don’t know 999* | |  |
| **MODULE E: MENTAL HEALTH INDICATOR** | | | | | | | | | |
| **Question #** | **Question** | | | | | | **Response** | | **Constraint** |
| ***Anxiety symptoms*** | | | | | | | | | |
| Note | READ THE FOLLOWING OUT LOUD TO THE RESPONDENT: “Listed below are symptoms or problems that people sometimes have. Please listen to each on carefully, and indicate how much the symptoms bothered you or distressed you in the last week, including today.” | | | | | |  | |  |
| E.1 | Suddenly scared for no reason - Read response options. | | | | | | *Not at all 0*  *A little 1*  *Quite a bit 2*  *Extremely 3*  *Refused 888* | |  |
| E.2 | Feeling fearful - Read response options. | | | | | | *Not at all 0*  *A little 1*  *Quite a bit 2*  *Extremely 3*  *Refused 888* | |  |
| E.3 | Faintness, dizziness or weakness - Read response options. | | | | | | *Not at all 0*  *A little 1*  *Quite a bit 2*  *Extremely 3*  *Refused 888* | |  |
| E.4 | Nervousness or shakiness inside - Read response options. | | | | | | *Not at all 0*  *A little 1*  *Quite a bit 2*  *Extremely 3*  *Refused 888* | |  |
| E.5 | Heart pounding or racing - Read response options. | | | | | | *Not at all 0*  *A little 1*  *Quite a bit 2*  *Extremely 3*  *Refused 888* | |  |
| E.6 | Trembling - Read response options. | | | | | | *Not at all 0*  *A little 1*  *Quite a bit 2*  *Extremely 3*  *Refused 888* | |  |
| E.7 | Feeling tense or keyed up - Read response options. | | | | | | *Not at all 0*  *A little 1*  *Quite a bit 2*  *Extremely 3*  *Refused 888* | |  |
| E.8 | Headaches – Read response options. | | | | | | *Not at all 0*  *A little 1*  *Quite a bit 2*  *Extremely 3*  *Refused 888* | |  |
| E.9 | Spell of terror or panic - Read response options. | | | | | | *Not at all 0*  *A little 1*  *Quite a bit 2*  *Extremely 3*  *Refused 888* | |  |
| E.10 | Feeling restless or can’t sit still - Read response options. | | | | | | *Not at all 0*  *A little 1*  *Quite a bit 2*  *Extremely 3*  *Refused 888* | |  |
| ***Depression symptoms*** | | | | | | | | | |
| E.11 | Feeling low in energy, slowed down - Read response options. | | | | | | *Not at all 0*  *A little 1*  *Quite a bit 2*  *Extremely 3*  *Refused 888* | |  |
| E.12 | Blaming yourself for things - Read response options. | | | | | | *Not at all 0*  *A little 1*  *Quite a bit 2*  *Extremely 3*  *Refused 888* | |  |
| E.13 | Crying easily - Read response options. | | | | | | *Not at all 0*  *A little 1*  *Quite a bit 2*  *Extremely 3*  *Refused 888* | |  |
| E.14 | Poor appetite - Read response options. | | | | | | *Not at all 0*  *A little 1*  *Quite a bit 2*  *Extremely 3*  *Refused 888* | |  |
| E.15 | Difficulty falling asleep, staying asleep - Read response options. | | | | | | *Not at all 0*  *A little 1*  *Quite a bit 2*  *Extremely 3*  *Refused 888* | |  |
| E.16 | Feeling hopeless about future - Read response options. | | | | | | *Not at all 0*  *A little 1*  *Quite a bit 2*  *Extremely 3*  *Refused 888* | |  |
| E.17 | Feeling blue - Read response options. | | | | | | *Not at all 0*  *A little 1*  *Quite a bit 2*  *Extremely 3*  *Refused 888* | |  |
| E.18 | Feeling lonely - Read response options. | | | | | | *Not at all 0*  *A little 1*  *Quite a bit 2*  *Extremely 3*  *Refused 888* | |  |
| E.19 | Feeling of being trapped or caught - Read response options. | | | | | | *Not at all 0*  *A little 1*  *Quite a bit 2*  *Extremely 3*  *Refused 888* | |  |
| E.20 | Worry too much about things - Read response options. | | | | | | *Not at all 0*  *A little 1*  *Quite a bit 2*  *Extremely 3*  *Refused 888* | |  |
| E.21 | Feeling no interest in things - Read response options. | | | | | | *Not at all 0*  *A little 1*  *Quite a bit 2*  *Extremely 3*  *Refused 888* | |  |
| E.22 | Feeling everything is an effort - Read response options. | | | | | | *Not at all 0*  *A little 1*  *Quite a bit 2*  *Extremely 3*  *Refused 888* | |  |
| E.23 | Feeling of worthlessness - Read response options. | | | | | | *Not at all 0*  *A little 1*  *Quite a bit 2*  *Extremely 3*  *Refused 888* | |  |
| ***WHO-5 Well-being index*** | | | | | | | | | |
| Note | READ THE FOLLOWING OUT LOUD TO THE RESPONDENT: “Please indicate for the 5 statements which is closest to how you have been feeling over the LAST 2 WEEKS.” Example: If you have felt cheerful and in good spirits more than half of the time during the last 2 weeks, indicate ‘more than half of the time.” | | | | | |  | |  |
| E.24 | I have felt cheerful and in good spirits - Read response options. | | | | | | *At no time (0 days) 1*  *Some of the time (1-3 days) 2*  *Less than a week (4-6 days) 3*  *More than a week (7-10 days) 4*  *Most of the time (11-13 days) 5*  *All the time (14 days) 6*  *Refused 888* | |  |
| E.25 | I have felt calm and relaxed - Read response options. | | | | | | *At no time (0 days) 1*  *Some of the time (1-3 days) 2*  *Less than a week (4-6 days) 3*  *More than a week (7-10 days) 4*  *Most of the time (11-13 days) 5*  *All the time (14 days) 6*  *Refused 888* | |  |
| E.26 | I have felt active and vigorous - Read response options. | | | | | | *At no time (0 days) 1*  *Some of the time (1-3 days) 2*  *Less than a week (4-6 days) 3*  *More than a week (7-10 days) 4*  *Most of the time (11-13 days) 5*  *All the time (14 days) 6*  *Refused 888* | |  |
| E.27 | I woke up feeling fresh and rested - Read response options. | | | | | | *At no time (0 days) 1*  *Some of the time (1-3 days) 2*  *Less than a week (4-6 days) 3*  *More than a week (7-10 days) 4*  *Most of the time (11-13 days) 5*  *All the time (14 days) 6*  *Refused 888* | |  |
| E.28 | My daily life has been filled with things that interest me Read response options. | | | | | | *At no time (0 days) 1*  *Some of the time (1-3 days) 2*  *Less than a week (4-6 days) 3*  *More than a week (7-10 days) 4*  *Most of the time (11-13 days) 5*  *All the time (14 days) 6*  *Refused 888* | |  |
| E.29 | Are you currently ill? | | | | | | *Yes 1*  *No 0*  *Refused 888*  *Don’t know 999* | |  |
| **MODULE F: ANIMAL HUSBANDRY & REPORTED WASTE MANAGEMENT** | | | | | | | | | |
| **Question #** | **Question** | | | | | | **Response** | | **Constraint** |
| Note | Ask the following question, and listen to the reply, then code answer accordingly. If the respondent does not specify how often the animal faeces is disposed of, follow with a probing question to ask if the disposal takes place on a daily basis or every few days/wkly. | | | | | |  | |  |
| F.1 | Last night, where did you keep your animals?  *Enumerator note: Select all that apply* | | | | | | *In the same room as children 1*  *Within the homestead, in the living room 2*  *In a pen or room outside of, but connected to the homestead (with ANY entrance INSIDE of the homestead) 3*  *In a pen or room outside of, but connected to the homestead (with NO entrance INSIDE of the homestead) 4*  *In a pen or room within the IMMEDIATE homestead compound, but not connected to the homestead 5*  *In the yard of the IMMEDIATE homestead compound 6*  *In the yard of the household compound (but OUTSIDE the immediate homestead compound) 7*  *Outside the household compound 8*  *Other 777*  *Don’t know 999* | | if (D.13+D.14+ D.15+D.16) >0 |
| F.2 | Ask: How did you dispose of the animal faeces/waste in your compound?  *Enumerator note: Select all that apply* | | | | | | *It’s left in the open in the home compound 0*  *DAILY, it’s placed (NOT BURIED) in a designated location WITHIN the home compound 1*  *DAILY, it’s BURIED in a designated location WITHIN the home compound 2*  *DAILY, it’s thrown somewhere OUTSIDE OF the home compound 3*  *DAILY, adding it directly to the field/plants as fertilizer 7*  *DAILY, fresh faeces are used to make dung patties 9*  *EVERY FEW DAYS/WEEKLY, it’s placed (NOT BURIED) in a designated location WITHIN the home compound 4*  *EVERY FEW DAYS/WEEKLY, it’s BURIED in a designated location WITHIN the home compound 5*  *EVERY FEW DAYS/WEEKLY, it’s thrown somewhere OUTSDIE OF the home compound 6*  *EVERY FEW DAYS/WEEKLY, adding it directly to the field/plants as fertilizer 8*  *Other 777*  *There is never any animal waste in the compound 666*  *Refused 888*  *Don’t know 999* | |  |
| F.3 | ASK: How do you dispose of solid was (e.g., rubbish)?  *Enumerator note: Ask the following question, and listen to the reply, then code answer accordingly. SELECT ALL THAT APPLY.* | | | | | | *It’s left out in the open INSIDE the home compound 0*  *It’s placed (not BURIED) in a designated location WITHIN the home compound 1*  *It’s BURIED in a designated location WITHIN the home compound 2*  *It’s thrown somewhere OUTSIDE of the home compound 3*  *It’s burned in a designated location INSIDE the home compound 4*  *It’s burned in a designated location OUTSIDE the home compound 5*  *It’s placed in a compost pin INSIDE the home compound 6*  *It’s placed in a compost pit OUTSIDE the home compound 7*  *Refused 888*  *Don’t know 999* | |  |
| **MODULE G: WASHING STATIONS** | | | | | | | | | |
| **Question #** | | | **Question** | | | | **Response** | | **Constraint** |
| G.1 | | | Does your household have any hand or facewashing stations? | | | | *Yes 1*  *No 0*  *Refused 888*  *Don’t know 999* | |  |
| note | | | Please show me all of your hand and facewashing stations.  *Enumerator note: Repeat all following questions for each hand and facewashing station.* | | | |  | | if G.1=1 |
| G.2 | | | OBSERVE: What are the components of the washing station:  *Enumerator note: Select all that apply* | | | | *Plastic basin 1*  *Wooden basin (gebeta) 2*  *Pitcher (maeqoreqoreia) 3*  *Dishes 4*  *Tippy tap (e.g., gourd, jerry can) 5*  *Other 777* | |  |
| G.3 | | | OBSERVE: Location of the handwashing/ facewashing station:  *Enumerator note: Select all that apply* | | | | *No specific place 0*  *Outside yard 1*  *Elsewhere in home or yard 2*  *Inside/within 10 paces of the latrine 4*  *Inside/within 10 paces of the kitchen/cooking place 3*  *Refused permission to see 888*  *Other 777* | |  |
| G.4 | | | OBSERVE: Whether this is a mobile or fixed-point washing station | | | | *Yes 1*  *No 0* | |  |
| G.5 | | | OBSERVE: Presence/ absence of water at handwashing/ facewashing stations | | | | *Water is absent from ALL washing stations 0*  *Water is present at some, but not all washing stations 1*  *Water is present at ALL washing stations 2* | |  |
| G.6 | | | OBSERVE: Presence/ absence of soap or soap substitute (e.g., soapy water, ash) at the washing stations | | | | *Soap/soap substitute is absent from ALL washing stations 0*  *Soap/soap substitute is present at some, but not all washing stations 1*  *Soap/soap substitute is present at ALL washing stations 2* | |  |
| **MODULE H: LATRINE INFROMATION MODULE** | | | | | | | | | |
| **Question #** | **Question** | | | | | | **Response** | | **Constraint** |
| **Latrine construction, operation & maintenance, and repair** | | | | | | | | | |
| Note | Ask, observe, and record the status of the household latrine facilities. Start by asking to be shown the MOST FREQUENTLY USED household latrine and fill the responses for that latrine, then ask to be SECOND MOST FREQUENTLY USED household latrine and fill the responses for that latrine, and so on. Say: “Now, will you please show me your latrine(s)?” | | | | | |  | | if A.7 !=0 |
| H.1 | OBSERVE: Did the respondent wear shoes to walk to the latrine? | | | | | | *Yes 1*  *No 0* | |  |
| H.2 | Who OWNS this household latrine? | | | | | | *Household owns the latrine THEMSELVES 1*  *Household SHARES ownership of the latrine 2*  *Another household owns the latrine 3*  *Refused 888*  *Don’t know 999* | |  |
| H.3 | Has any member of your household use this latrine for 3 or more days during the LAST 7 days, including today? | | | | | | *Yes 1*  *No 0*  *Refused 888*  *Don’t know 999* | |  |
| H.4 | During the LAST 7 days, how many household have USED this household latrine?  *Enumerator note: Enter 999 if respondent doesn't know or can't recall. Enter 888 if respondent refused to answer.* | | | | | | *__ __ households* | |  |
| H.5 | During the LAST 7 days, **NOT INCLUDING your** household members, how many people used this household latrine?  *Enumerator note: This question is asking about NON-HOUSEHOLD MEMBERS who use this latrine! Enter 999 if respondent doesn't know or can't recall. Enter 888 if respondent refused to answer.* | | | | | | *__ __ people* | |  |
| H.6 | Who chose the design for this latrine? | | | | | | *A member of this household 1*  *An NGO 2*  *Government, including HEW 3*  *Relative, friend, or neighbor 4*  *Paid contractor 5*  *Refused 888*  *Don’t know 999* | |  |
| H.7 | During the last 7 days, including today, how many times was this latrine cleaned?  *Enumerator note: Enter 999 if respondent doesn't know or can't recall. Enter 888 if respondent refused to answer.* | | | | | | __ __ times | |  |
| H.8 | In what month and year was the ORIGINAL latrine construction completed? | | | | | | *Month: _____________*  *Year: __ __ __ __* | |  |
| H.9 | What is the MAIN reason you decided to construct the latrine at that time? | | | | | | *Self-motivated 1*  *HEW or other government official urged us to do so 2*  *Relative, friend, or neighbor urged us to do so 3*  *Other 777*  *Refused 888*  *Don’t know 999* | |  |
| H.10 | Did someone from your household contribute labor for the ORIGINAL construction of the latrine? | | | | | | *Yes 1*  *No 0*  *Refused 888*  *Don’t know 999* | |  |
| H.11 | Was this person trained in latrine construction? | | | | | | *No, no training at all 0*  *Yes, but only informal training (by a neighbor or friend) 1*  *Yes, formal training provided by a professional mason/carpenter 2*  *Refused 888*  *Don’t know 999* | | if H.10=1 |
| H.12 | Are you satisfied, dissatisfied or neither satisfied nor dissatisfied in the latrine as a place for you and your household to defecate? | | | | | | *Dissatisfied 0*  *Satisfied 1*  *Neither satisfied nor dissatisfied 2*  *Refused 888*  *Don’t know 999* | |  |
| H.13 | Have you ever fixed anything that became broken, damaged, or worn out on this latrine since its original construction (i.e., has the latrine been repaired since its original construction)? | | | | | | *Yes 1*  *No 0*  *Refused 888*  *Don’t know 999* | |  |
| H.14 | Have you added or improved anything on this latrine to upgrade it since its original construction (i.e. has the latrine been upgraded or improved upon since its original construction)? | | | | | | *Yes 1*  *No 0*  *Refused 888*  *Don’t know 999* | |  |
| H.15 | Is your latrine facility working (operating) correctly now? | | | | | | *Yes 1*  *No 0*  *Refused 888*  *Don’t know 999* | |  |
| H.16 | Do you consider your latrine facility, as it is today, fully constructed? | | | | | | *Yes 1*  *No 0*  *Refused 888*  *Don’t know 999* | |  |
| H.17 | What is the below ground structure of the facility? | | | | | | *Unlined pit 0*  *Lined pit beneath latrine 1*  *Lined pit offset from the latrine 2*  *Don’t know 999* | |  |
| H.18 | How many pits does this latrine have?  **Number has to be between 1 and 5* | | | | | | *__ pits* | |  |
| H.19 | How deep is this latrine pit?  *If there are multiple pits, enter information for the pit currently in use* | | | | | | *No actual latrine pit (only superstructure) 0*  *Less than 3 metres 1*  *3 metres or more 2*  *Refused 888*  *Don’t know 999* | |  |
| ***Sludge management*** | | | | | | | | | |
| H.20 | Has this latrine’s pit ever filled completely? | | | | | | *Yes 1*  *No 0*  *Refused 888*  *Don’t know 999* | |  |
| H.21 | Did you empty the latrine pit? | | | | | | *Yes 1*  *No 0*  *Refused 888*  *Don’t know 999* | | if H.20=1 |
| H.22 | What was done with the sludge? | | | | | | *Spread on the field as fertilizer 0*  *Dumped in the open/bush 1*  *Dumped in the river/pond/canal 2*  *Emptied into new hole or buried (including filling in the pit with earth) 3*  *Other 777*  *Refused 888*  *Don’t know 999* | | if H.21=1 |
| ***Latrine spot checks: structure, functionality, cleanliness***  *Structural status of household latrine (Ask or observe):* | | | | | | | | | |
| H.23 | OBSERVE: Location of latrine: | | | | | | *In/attached to own dwelling 1*  *In own compound (but not in the household dwelling) 2*  *In/attached to neighbour’s dwelling 3*  *In neighbour’s compound (but not in the neighbour’s household dwelling) 4*  *Refused to allow observation 888* | |  |
| H.24 | OBSERVE: Type of latrine: | | | | | | *Pit latrine with rudimentary platform (platform made with wood, and either NOT plastered, or poorly plastered so the pit is not properly sealed) 1*  *Pit latrine with solid platform of wood/logs and mud/dung plaster that properly seals the pit 3*  *Hanging toilet/latrine 2*  *Pit latrine with concrete slab 4*  *Pit latrine with concrete slab & water seal 5*  *Composting toilet (toilet that ensures separation of urine, water, and extreta) 6*  *Ventilated improved pit (VIP) latrine with concrete slab or solid platform that seals the pit 7*  *Flush/pour flush to pit 8*  *Flush/pour flush to septic tank 9*  *Flush/pour flush to elsewhere 10*  *Other 777*  *Refused to allow observation 888* | |  |
| H.25 | OBSERVE: Does the latrine require any obvious repairs? | | | | | | *Yes 1*  *No 0*  *Refused 888*  *Don’t know 999* | |  |
| H.26 | OBSERVE: Is this latrine “serviceable”, meaning the pit is not overflowing, and the floor provides a solid foundation over the latrine pit?  *Enumerator note: For the purposes of this study, a "serviceable" latrine is one that has a latrine drop hole and pit that is not overflowing, with a floor that provides a solid foundation for use.* | | | | | | *Non-serviceable 0*  *Serviceable 1*  *Cannot observe (e.g., door locked) 2*  *Refused to allow observation 888* | |  |
| H.27 | OSBERVE: Status of reserve pit covering: | | | | | | *Pit was fully open/no cover 0*  *Cover was partially open/broken 1*  *Pit was visible but FULLY covered 2*  *Pit buried and not visible 3*  *Other 777* | | if H.26>1 |
| H.28 | OBSERVE: What material are the walls of the latrine made of?  *Select all that apply* | | | | | | *No superstructure 0*  *Mud/dung/bamboo/wood/straw 1*  *Bamboo/wood/straw (no mud/dung) 2*  *Cloth/curtain 3*  *Plastic sheeting 4*  *Tin/metal sheet 5*  *Other material 777* | |  |
| H.29 | OBSERVE: Height of latrine super-structure: | | | | | | *No superstructure 0*  *Less than full-height (i.e., less than 2 metres) when standing inside the latrine 1*  *Full-height (i.e., 2 metres or more) when standing in the latrine 2*  *Other 777* | |  |
| H.30 | OBSERVE: Material of latrine door:  *Select all that apply* | | | | | | *No door present 0*  *Bamboo/wood 1*  *Cloth/curtain 2*  *Plastic sheeting 3*  *Tin/metal sheet 4*  *Other material 777* | |  |
| H.31 | OBSERVE: Does door close completely? | | | | | | *Yes 1*  *No 0*  *Don’t know (can’t observe/assess) 999* | | if H.30 !=0 |
| H.32 | OBSERVE: Material of latrine roof  *Select all that apply* | | | | | | *No roof present 0*  *Thatch/grass/plastic sheeting 1*  *Corrugated tin 2*  *Tiles/tally 3*  *Concrete 4*  *Other material 777* | |  |
| H.33 | OBSERVE: Does the latrine roof fully cover the latrine (i.e., no holes or gaps to keep the rain out)? | | | | | | *Yes 1*  *No 0* | |  |
| H.34 | OBSERVE: Material of latrine floor around pit:  *Select all that apply* | | | | | | *Mud/earth/stone 1*  *Bamboo/wood 2*  *Plastic sheeting 3*  *Concrete only 4*  *Concrete & tiles 5*  *Other material 777* | |  |
| H.35 | OBSERVE: Is the latrine floor a smooth and cleanable surface?  *Enumerator note: In order to be a smooth and cleanable surface, the floor must be made of a material that can be sanitized, such as non-porous cement slab or tiles (ceramic or plastic)* | | | | | | *Yes 1*  *No 0*  *Don’t know (can’t observe/assess) 999* | |  |
| ***Other spot check indicators*** | | | | | | | | | |
| H.36 | OBSERVE: Presence of stagnant water (water log) over the floor, latrine slab? | | | | | | *Yes 1*  *No 0*  *Don’t know (can’t observe/assess) 999* | |  |
| H.37 | OBSERVE: Evidence of discoloration of slab or floor (e.g., yellow, green or brown)? | | | | | | *Yes 1*  *No 0*  *Don’t know (can’t observe/assess) 999* | |  |
| H.38 | OBSERVE: Presence of flies in latrine? | | | | | | *Yes 1*  *No 0*  *Don’t know (can’t observe/assess) 999* | |  |
| H.39 | OBSERVE: Presence of hole cover in the latrine? | | | | | | *Yes 1*  *No 0*  *Don’t know (can’t observe/assess) 999* | |  |
| H.40 | OBSERVE: is the drop hole cover currently situated over the latrine drop hole (i.e., fully covering the drop hole)? | | | | | | *Yes 1*  *No 0*  *Don’t know (can’t observe/assess) 999* | | if H.38=1 |
| H.41 | OBSERVE: Presence of cleaning agents for washing latrine (inside or within 10 paces of the latrine)? | | | | | | *Yes 1*  *No 0*  *Don’t know (can’t observe/assess) 999* | |  |
| H.42 | OBSERVE: Presence of faeces on floor/slab or other place in the latrine aside from the pit? | | | | | | *Yes 1*  *No 0*  *Don’t know (can’t observe/assess) 999* | |  |
| H.43 | OBSERVE: Evidence latrine is used for storage or other non-sanitation-related purpose? | | | | | | *Yes 1*  *No 0*  *Don’t know (can’t observe/assess) 999* | |  |
| H.44 | OBSERVE: Presence of well-worn path to latrine? | | | | | | *Yes 1*  *No 0*  *Don’t know (can’t observe/assess) 999* | |  |
| H.45 | OBSERVE: Presence of fresh faeces in the pit or on the floor inside the latrine? | | | | | | *Yes 1*  *No 0*  *Don’t know (can’t observe/assess) 999* | |  |
| H.46 | OBSERVE: Is the pit that is in use full or close to being full (i.e., you can visibly see waste within the top 0.3 meter of the latrine pit, or the pit is actively overflowing)? | | | | | | *Yes 1*  *No 0*  *Don’t know (can’t observe/assess) 999* | |  |
| H.47 | OBSERVE: Presence of items for anal cleansing, wiping in, near toilet (e.g., paper, leaves, straw, wood, maize cobb, water)? | | | | | | *Yes 1*  *No 0*  *Don’t know (can’t observe/assess) 999* | |  |
| H.48 | OBSERVE: Presence of odour from stools or urine in the latrine? | | | | | | *Yes 1*  *No 0*  *Don’t know (can’t observe/assess) 999* | |  |
| H.49 | OBSERVE: Presence of leaves, spider webs, rubbish, other dirt in or on latrine drop hole? | | | | | | *Yes 1*  *No 0*  *Don’t know (can’t observe/assess) 999* | |  |
| H.50 | OBSERVE: Wet latrine floor? | | | | | | *Yes 1*  *No 0*  *Don’t know (can’t observe/assess) 999* | |  |
| H.51 | OBSERVE: Presence of water available inside or within 10 paces of the latrine for handwashing? | | | | | | *Yes 1*  *No 0*  *Don’t know (can’t observe/assess) 999* | |  |
| H.52 | OBSERVE: Presence of cleansing agent (soap, detergent, soapy water) inside or within 10 paces of the latrine for handwashing? | | | | | | *Yes 1*  *No 0*  *Don’t know (can’t observe/assess) 999* | |  |
| H.53 | OBSERVE: Water available inside or within 10 paces of the latrine for flushing or self-cleansing? | | | | | | *Yes 1*  *No 0*  *Don’t know (can’t observe/assess) 999* | |  |
| H.54 | OBSERVE: Enter the number of minutes it takes to walk FROM the latrine to the FRONT door of the domestic living space within the homestead.  *Enumerator note: Enter 1 if less than 1 minute. Enter 888 if respondent refused to allow observation.* | | | | | | __ __ minutes | |  |
| **MODULE I: WASTE OBSERVATIONS** | | | | | | | | | |
| **Question #** | **Question** | | | | | | **Response** | | **Constraint** |
| note | Walk around the household compound (within and around the outside, if possible), the latrine, and any walkways within the compound as well as walkways that are access points into the household in search of human faeces. | | | | | |  | |  |
| I.1 | OBSERVE: Is there evidence of open defecation (i.e., human faeces) in or near the household compound? | | | | | | *No 0*  *Yes, within the household compound 1*  *Yes, near but outside of the household compound 2* | |  |
| I.2 | OBSERVE: How does the household appear to be disposing of the animal faeces/waste?  *Enumerator note: Observe the household compound, and look for any evidence of animal faeces. If the respondent indicates they dispose of the animal’s faeces within the home compound (EITHER placing OR burying the faeces in a designated location), ask to be shown the disposal site to confirm whether the waste is being placed out in the open or buried.* | | | | | | *It’s left out in the open in the home compound 0*  *It’s PLACED in a designated location WITHIN the home compound 1*  *It’s BURIED in a designated location WITHIN the home compound 2*  *It’s either placed or buried OUTSIDE of the home compound (i.e., no animal faeces present in the home compound) 3* | |  |
| I.3 | OBSERVE: How does the household appear to be disposing of solid waste?  *Enumerator note: Observe the household compound, and look for any evidence of solid waste/rubbish. If the respondent indicates they dispose of solid waste within the home compound, ask to be shown the disposal site to confirm whether the waste is disposed in the open or buried.* | | | | | | *It’s left out in the open in the home compound 0*  *It’s PLACED in a designated location WITHIN the home compound 1*  *It’s BURIED in a designated location WITHIN the home compound 2*  *It’s either placed/buried OUTSIDE Of the home compound (i.e., no solid waste present in the home compound) 3* | |  |
| I.4 | OBSERVE: Is there animal faeces present in the compound? | | | | | | *Yes 1*  *No 0* | |  |
| **MODULE J: INFORM HOUSEHOLD WE WILL BE BACK** | | | | | | | | | |
| **Question #** | **Question** | | | | | | **Response** | | **Constraint** |
| Add info | Enumerator: Please provide details regarding any issues or information related to this survey that we should know about. | | | | | |  | |  |
| Geopoint | Household geocoordinate: | | | | | |  | |  |
| End | What was the result of the survey? | | | | | | *No household member between the ages of 1-9 years 1*  *No eligible respondent available 2*  *Eligible respondent refused to consent 3*  *Partially completed – respondent refused to complete the survey 4*  *Finished to completion 5* | |  |
